# Neutrophil-derived Activin-A moderates their pro-NETotic activity and attenuates collateral tissue damage caused by Influenza A virus infection

**DOI:** 10.3389/fimmu.2024.1302489

**Published:** 2024-02-26

**Authors:** Georgios Divolis, Evgenia Synolaki, Athanasia Doulou, Ariana Gavriil, Christina C. Giannouli, Anastasia Apostolidou, Martyn L. Foster, Martin M. Matzuk, Panagiotis Skendros, Ioanna-Evdokia Galani, Paschalis Sideras

**Affiliations:** ^1^ Center for Clinical, Experimental Surgery and Translational Research, Biomedical Research Foundation Academy of Athens, Athens, Greece; ^2^ Experimental Pathology Consultant, London, United Kingdom; ^3^ Department of Pathology & Immunology, Baylor College of Medicine, Houston, TX, United States; ^4^ Center for Drug Discovery, Baylor College of Medicine, Houston, TX, United States; ^5^ Laboratory of Molecular Hematology, Department of Medicine, Democritus University of Thrace, Alexandroupolis, Greece; ^6^ First Department of Internal Medicine, University Hospital of Alexandroupolis, Democritus University of Thrace, Alexandroupolis, Greece

**Keywords:** Activin-A, neutrophils, Influenza A, inflammation, NETs, lung, ALK4

## Abstract

**Background:**

Pre-neutrophils, while developing in the bone marrow, transcribe the *Inhba* gene and synthesize Activin-A protein, which they store and release at the earliest stage of their activation in the periphery. However, the role of neutrophil-derived Activin-A is not completely understood.

**Methods:**

To address this issue, we developed a neutrophil-specific Activin-A-deficient animal model (*S100a8-Cre/Inhba* ^fl/fl^ mice) and analyzed the immune response to Influenza A virus (IAV) infection. More specifically, evaluation of body weight and lung mechanics, molecular and cellular analyses of bronchoalveolar lavage fluids, flow cytometry and cell sorting of lung cells, as well as histopathological analysis of lung tissues, were performed in PBS-treated and IAV-infected transgenic animals.

**Results:**

We found that neutrophil-specific Activin-A deficiency led to exacerbated pulmonary inflammation and widespread hemorrhagic histopathology in the lungs of IAV-infected animals that was associated with an exuberant production of neutrophil extracellular traps (NETs). Moreover, deletion of the Activin-A receptor ALK4/ACVR1B in neutrophils exacerbated IAV-induced pathology as well, suggesting that neutrophils themselves are potential targets of Activin-A-mediated signaling. The pro-NETotic tendency of Activin-A-deficient neutrophils was further verified in the context of thioglycollate-induced peritonitis, a model characterized by robust peritoneal neutrophilia. Of importance, transcriptome analysis of Activin-A-deficient neutrophils revealed alterations consistent with a predisposition for NET release.

**Conclusion:**

Collectively, our data demonstrate that Activin-A, secreted by neutrophils upon their activation in the periphery, acts as a feedback mechanism to moderate their pro-NETotic tendency and limit the collateral tissue damage caused by neutrophil excess activation during the inflammatory response.

## 1 Introduction

Influenza A virus (IAV) infection may trigger life-threatening complications, including pneumonia and acute respiratory distress syndrome (ARDS), that account for approximately half a million deaths every year (1, 2). A hallmark of acute inflammation following IAV infection is the robust recruitment of neutrophils into the lungs (3, 4). These cells represent the most abundant white blood cell type in human peripheral blood and the first line of host defense against infection, trauma, or other disturbances of homeostasis (5–7). Upon activation, neutrophils degranulate and release a plethora of antimicrobial and pro-inflammatory proteins, and phagocytize pathogens, which they destroy through cytosolic free radicals and enzymes (8). They also respond through the formation of neutrophil extracellular traps (NETs). NETs are networks of extracellular chromatin fibers that are usually citrullinated and decorated with antimicrobial peptides and enzymes, such as neutrophil elastase, defensins, and myeloperoxidase (MPO) (9–11). Production of NETs is driven by a programmed form of cell death named NETosis. Both protective and detrimental properties have been attributed to NETs in the context of IAV infection (4, 12, 13). While NETs can trap and inactivate IAV particles (14), their overproduction or ineffective clearance compromises alveolar integrity, leading to widespread lung injury that correlates with the severity of the disease (13, 15).

Neutrophil infiltration and NETs have been implicated in the pathophysiology of several other respiratory immunoinflammatory diseases, including allergic airway inflammation (16, 17), interstitial lung disease (18, 19), ARDS (20, 21), and Coronavirus disease 2019 (COVID-19) (22, 23). Interestingly, another common characteristic of the aforementioned disorders is the deregulation of the Activin-A/Follistatin system, suggesting its possible involvement in the neutrophil-driven pathophysiology (24–29).

Activin-A is a member of the Transforming Growth Factor β (TGFβ) superfamily (30, 31). The active protein is composed of two disulfide-linked inhibin βA subunits. It has been ascribed context-dependent, pro-inflammatory, anti-inflammatory, and tissue repair/remodeling properties (26, 32, 33). Activin-A is produced following inflammatory stimulation by a number of cell types, including monocytes (34, 35), macrophages (36, 37), dendritic cells (38), T and B lymphocytes (39, 40), and natural killer (NK) cells (41). Neutrophils have also been identified as a source of Activin-A in the human asthmatic airway upon allergen challenge (42). Interestingly, pre-neutrophils, while developing in the bone marrow, activate transiently the *Inhba* gene and synthesize Activin-A protein (43). This preformed Activin-A is released in the bloodstream during the earliest stages of neutrophil activation, as exemplified in animal models of lipopolysaccharide-induced inflammation (44–46). Human peripheral blood neutrophils store mature preformed Activin-A as well, which they release following TNF-α stimulation *in vitro* (47).

The precise role of Activin-A which is either pre-loaded in neutrophils during their early development or synthesized *de novo* during their activation in the periphery has not been clarified yet. To address this, we developed a mouse model in which Activin-A was selectively depleted from neutrophils and assessed neutrophil function in the context of a severe respiratory inflammatory response induced by IAV infection.

Our findings demonstrate that neutrophil-derived Activin-A moderates the pro-NETotic tendency of activated neutrophils, thereby describing a novel mechanism for the control of neutrophil-mediated inflammation and collateral tissue damage.

## 2 Materials and methods

### 2.1 Animals

*S100a8*-Cre-ires/eGFP mice (48), hereafter called *S100a8*-Cre, were purchased from The Jackson Laboratory. *Inhba* ^fl/fl^ mice were genotyped as described (49). *Acvr1b* ^fl/fl^ (50) and Gt(ROSA)26Sor^tm4(ACTB-tdTomato,-EGFP)Luo^ mice (51), herein called Rosa-Tomato^fl/fl^, were kindly provided by Dr. G. Xanthou and Dr. A. Klinakis [Biomedical Research Foundation of the Academy of Athens (BRFAA), Athens, Greece], respectively. *S100a8*-Cre were crossed to *Inhba* ^fl/fl^ to generate *S100a8*-Cre/*Inhba* ^fl/fl^, to *Acvr1b* ^fl/fl^ to generate *S100a8*-Cre/*Acvr1b* ^fl/fl^, and to Rosa-Tomato^fl/fl^ to generate *S100a8*-Cre/Rosa-Tomato^fl/fl^ mice, all maintained in C57BL/6 background. *S100a8*-Cre, *Inhba* ^fl/fl^, *Acvr1b* ^fl/fl^, and Rosa-Tomato^fl/fl^ mice were used as controls. In all experiments, 10-12-week-old female mice, housed at the animal house facility of the BRFAA as reported (52), were used.

### 2.2 Infection with Influenza A virus

Purified A/PR/8/34 (H1N1) Influenza virus was purchased from Charles River Laboratories. Infectious viral load was determined in Madin-Darby canine kidney cells, kindly provided by Dr. R. Walton (Imperial College London, UK), as previously described (53). Mice were anesthetized as reported (53, 54), infected intranasally with a single non-lethal dose of 50 plaque-forming units (pfu) of IAV, supplied in a volume of 40 μl sterile phosphate-buffered saline (PBS), and monitored daily for body weight loss. Tissues were processed for molecular or histological analyses at 3, 5, 8, 15, and 35 days p.i. For survival experiments, mice were infected with a single dose of 500 pfu of IAV and monitored daily for mortality. Viral load was determined by the detection of the IAV *Ns1* RNA in the right lung lobes of IAV-infected animals, using RT-qPCR, and primers published elsewhere (55).

### 2.3 Lung mechanics and histology

Lung function was evaluated by monitoring static compliance and total lung resistance in anesthetized mice, as previously reported (25, 53).

Histological analysis of lungs from *S100a8*-Cre/*Inhba* ^fl/fl^, *S100a8*-Cre/*Acvr1b* ^fl/fl^, and respective control animals was performed in paraffin-embedded tissues, as previously described (25, 53). Three-point five µm thick sections, performed in the sagittal plane at the level of the respiratory tree, were stained with hematoxylin and eosin (H&E) or used for immunofluorescence analysis. Further histological analysis of lungs from *S100a8*-Cre/Rosa-Tomato^fl/fl^ and respective control animals was performed in cryosections, as previously reported (56). Processed tissues were embedded in cryomatrix embedding resin (Shandon Cryomatrix, 6769006) and sectioned in a cryostat (Leica CM3050S) through the sagittal plane at 6 μm increments.

### 2.4 Immunofluorescence analysis

Immunostaining of either paraffin or cryostat sections was performed as described (53, 57). The following primary: rabbit anti-histone H3 (Abcam, ab1791, 1:300), rabbit anti-citrullinated histone H3 (anti-cit-H3; Abcam, ab5103, 1:200), goat anti-MPO (R&D Systems, AF3667, 1:100), rat anti-Ly6G (Bio-X-Cell, BE0075-1, 1:800), and secondary antibodies, raised in donkey: anti-rabbit Alexa 488 (Jackson ImmunoResearch, 711-546-152, 1:200), anti-rat Alexa 488 (Jackson ImmunoResearch, 712-586-150, 1:200), anti-goat Alexa 647 (Jackson ImmunoResearch, 705-605-147, 1:200), and anti-goat Alexa 594 (Jackson ImmunoResearch, 705-586-147, 1:200) were used. 4′,6-diamidino-2-phenylindole (DAPI) dihydrochloride (Calbiochem, 268298, 2.5 μg/ml) was used for nuclear staining, and sections were mounted with a fluorescence mounting medium (Dako, S3023). Images were captured with a Leica TCS-SP5II confocal microscope (Leica Microsystems, Germany) and were analyzed using Adobe Photoshop CS6 and Fiji/ImageJ v2.0.0 software. Quantification of fluorescence intensity of histone H3 and cit-H3 in lung sections was performed using Fiji/ImageJ v2.0.0 software. In brief, confocal immunofluorescence images at 20x magnification were used, and the “mean gray value” was selected to measure mean fluorescence intensity, which was normalized over the area. A total of 25 fields/group were measured.

### 2.5 Bronchoalveolar lavage fluid analysis and differential cell counting

Bronchoalveolar lavage fluid (BAL) collection was performed as previously reported (25, 53). In brief, BAL was obtained from the whole mouse lung with two gentle 0.5 ml PBS lavages using a tracheal cannula, and then samples were centrifuged (2000 rpm, 10 min). Cell pellet smears were prepared on glass slides via cytospin centrifugation (600 rpm, 3 min), stained with May-Grünwald-Giemsa, and differential cell counting for neutrophils, macrophages, eosinophils, and lymphocytes was performed. BAL supernatants were analyzed for CXCL1 (KC), CXCL9 (MIG), CXCL10 (IP-10), G-CSF, IFNγ, IL-5, IL-6, IL-10, LIX, MCP-1 (CCL2), MIP-1α (CCL3), MIP-1β (CCL4), RANTES, and TNF-α presence using Milliplex technology on a Luminex 200 System according to the manufacturer’s instructions (Merck Millipore). Sandwich enzyme-linked immunosorbent assay (ELISA) kits were used for the detection of Activin-A (AnshLabs, AL-110) and mouse IgM levels (ThermoFisher Scientific, 88-50470) in BAL supernatants, according to the manufacturer’s instructions. The total protein concentration in the same samples was determined using the Bradford assay (58).

### 2.6 Flow cytometry and cell sorting

Lung digestion and staining procedure for flow cytometry have been described earlier (53, 56). Stained cells were acquired on a FACS Aria IIu (BD Biosciences) and sorted for neutrophils (CD45^+^CD11b^+^Ly6G^+^), alveolar macrophages (CD45^+^CD11c^+^SiglecF^+^), Ly6C^-^ monocytes (CD45^+^CD11c^-^CD11b^+^Ly6C^-^Ly6G^-^), inflammatory monocytes (CD45^+^CD11c^-^CD11b^+^Ly6C^+^Ly6G^-^), CD8^+^ T cells (CD45^+^CD3^+^CD8^+^), CD4^+^ T cells (CD45^+^CD3^+^CD4^+^), NK T cells (CD45^+^NK1.1^+^CD3^+^), NK cells (CD45^+^NK1.1^+^CD3^-^), conventional dendritic cells (CD45^+^CD11c^+^MHC-II^+^, expressing either CD11b or CD103), plasmacytoid dendritic cells (CD45^+^CD45R/B220^+^SiglecH^+^), and B cells (CD45^+^CD45R/B220^+^) (**Supplementary Figure 1**). Sorted cells were centrifuged (1400 rpm, 10 min, 4°C), and cell pellets were further processed for RNA isolation. A similar staining protocol was followed with isolated peritoneal cells as starting material. Data were analyzed with BD FACSDiva software (BD Biosciences). The antibodies used for flow cytometry and cell sorting are listed in **Supplementary Table 1**.

### 2.7 Thioglycollate-induced peritonitis and peritoneal cell analysis

Control and *S100a8*-Cre/*Inhba* ^fl/fl^ mice were intraperitoneally injected with 1 ml sterile 4% thioglycollate medium (TG; BD Difco, 211716) in dH_2_O, and peritoneal cells were collected with two gentle lavages of 5 ml ice-cold PBS, six hours post-injection. Peritoneal cells were used in the following experimental settings: (i) flow cytometric analysis for estimation of neutrophil and T cell numbers, (ii) *in vitro* NETosis evaluation, by culturing cells onto coverslips in RPMI 1640 medium (ThermoFisher Scientific, 61870) supplemented with 2% fetal bovine serum (FBS; ThermoFisher Scientific, 10500) for 0.5, 3, 6, 9, and 12 hours. Cultured cells were rinsed in PBS, fixed with 4% PFA (30 min, RT), and stained with an anti-cit-H3 antibody (Abcam, 1:200). The *in vitro* NETosis was evaluated by counting the cit-H3^+^ cells using Fiji/ImageJ v2.0.0 software. In brief, the specimens were photographed at 40x magnification, and the Cell Counter plugin was used to count the total number of cells by analyzing the area of DAPI^+^ nuclei per image. The pro-NETotic neutrophils and NETs were counted manually. A total of 1000 cells were counted per sample, (iii) flow cytometric analysis following staining with specific fluorescent probes. In brief, 3x10^6^ cells/sample were stained against surface markers CD45, CD11b, and Ly6G, as described earlier, washed in PBS supplemented with 1mM ethylenediaminetetraacetic acid (EDTA; Sigma-Aldrich, E7889) and 1% bovine serum albumin (BSA; Sigma-Aldrich, A9647), and centrifuged (1400 rpm, 10 min, 4°C). Cell pellets were resuspended in 1 ml Hanks’ Balanced Salt Solution (ThermoFisher Scientific, 24020117) supplemented with 0.5% FBS with either of the following fluorescent metabolic probes: 20mM 2′,7′–dichlorofluorescin diacetate (DCFDA; Sigma-Aldrich, D6883), 2mM MitoSox Red (ThermoFisher Scientific, M36007), 25nM tetramethyl rhodamine ethyl ester (TMRE) perchlorate (Sigma-Aldrich, 87917), following 30 min incubation at 37°C, 5% CO_2_, or 5mM 4,5-diaminofluorescein (DAF-2; Cayman Chemicals, 85160), for 1 hour at 37°C, 5% CO_2_. At the end of the incubation, cells were washed twice with 2 ml PBS supplemented with 1mM EDTA and 1% BSA, centrifuged (1400 rpm, 10 min, 4°C), and resuspended in PBS, prior to flow cytometric analysis, (iv) peritoneal neutrophil purification by cell sorting, and further processing for transcriptome analysis.

### 2.8 Isolation and *in vitro* culture of human peripheral blood neutrophils

For *in vitro* NETosis evaluation, peripheral blood neutrophils were isolated from EDTA-anticoagulated blood of healthy individuals by Histopaque (Sigma-Aldrich, 1077 and 1119) double-gradient density centrifugation (900g, 35 min, RT), as previously described (59). Isolated human neutrophils were cultured onto coverslips in RPMI 1640 medium (ThermoFisher Scientific) supplemented with 2% human serum (Sigma-Aldrich, H6914), and treated with 10μg/ml ActRIIB-Fc, a fusion protein containing the extracellular domain of the Activin type IIB receptor (ActRIIB/ACVR2B) and the Fc fragment of human IgG1 (25), or human IgG1-Fc as control, for 1, 1.5 and 2.5 hours at 37°C, 5% CO_2_. Then, human neutrophils were processed for immunostaining for cit-H3, as described earlier for mouse peritoneal cells.

### 2.9 RNA isolation, cDNA synthesis, and RT-qPCR

For mRNA expression analysis, the right lung lobes of mice were excised, immediately snap-frozen in liquid nitrogen, and stored at -80°C until used. Bone marrow cells were flushed from the femora and tibiae of mice using 20-30 ml ice-cold RPMI 1640 medium (ThermoFisher Scientific). Suspensions were passed through a 40 μm cell strainer and then centrifuged (1400 rpm, 10 min, 4°C). Cells were washed in ice-cold PBS, and red blood cell (RBC) lysis was performed with the RBC lysis buffer (Abcam, ab204733), according to the manufacturer’s instructions. Then, cells were washed with 10 ml ice-cold PBS supplemented with 2% FBS, centrifuged (1400 rpm, 10 min, 4°C), and cell pellets were used for subsequent analysis.

RNA isolation from frozen lungs or purified cells, followed by cDNA synthesis, was performed as previously described (56, 57). Primer pairs' design (**Supplementary Table 2**), and RT-qPCR reactions and parameters have been previously described (57). Data were collected using the LightCycler^®^ 96 System (Roche) and analyzed with the LightCycler^®^ 96 SW 1.1 software. Relative levels of mRNA expression were normalized to *Gapdh* and calculated according to the 2^–ΔΔCT^ method.

### 2.10 RNA sequencing and bioinformatics analysis

One μg of total RNA was used for the preparation of cDNA libraries, as previously described (57, 60). Sequencing was performed in a single-end manner at the Greek Genome Center (BRFAA, Athens, Greece), using the NextSeq 500/550 75c kit (Illumina, 20024906).

Raw sequence data were uploaded to the Galaxy web platform (61), and standard tools of the public server “usegalaxy.org” were used for subsequent analysis, as previously described (60). HISAT2 (v2.2.1+galaxy0) was applied for the alignment of trimmed reads to the mouse GRCm37/mm9 genome assembly from the Genome Reference Consortium.

Bioinformatics analysis was performed using the Ingenuity^®^ Pathway Analysis software (IPA^®^, Qiagen), the GeneCodis4 web-based tool (62), and the STRING database v11.5 (63). Cutoff values for differentially expressed genes were baseMean >100 and adjusted p-value (false discovery rate, FDR) <0.05. Gene set enrichment analysis (GSEA) was performed using the GSEA software (University of California, San Diego & Broad Institute, USA) (64). Briefly, normalized counts generated with the DESeq2 algorithm (v2.11.40.6+galaxy1) and annotated gene sets from the Mouse Molecular Signatures Database (Mouse MSigDB v2023.1) were used as inputs. Gene sets were ranked by taking the -log10 (p-value) and signed as positive or negative based on the direction of fold change, followed by pre-ranked analysis using the default settings (1000 permutations, min and max term size of 15 and 500, respectively).

Heatmaps and volcano plots were generated using the Morpheus, https://software.broadinstitute.org/morpheus (Broad Institute, USA), and the GraphPad Prism software (v8.4.3, San Diego, CA, USA), respectively.

### 2.11 Statistical analysis

Data are expressed as the mean ± standard error of the mean (SEM). Statistical analysis was performed with the GraphPad Prism software (v8.4.3, San Diego, CA, USA), using the nonparametric Mann-Whitney test for comparison between two groups and one-way analysis of variance (ANOVA) followed by Bonferroni’s *post hoc* test for comparison between three (or more) groups. One asterisk (*) corresponds to a statistical significance of p < 0.05, two asterisks (**) to p < 0.01, and three asterisks (***) to p < 0.001.

## 3 Results

### 3.1 Conditional Activin-A deletion from neutrophils results in severe lung damage upon Influenza A virus infection

The *S100a8/Mrp8*-Cre mice were used to drive neutrophil-specific, Cre-mediated deletion of floxed alleles. To confirm the specificity of this system, *S100a8*-Cre were initially crossed with Rosa-Tomato^fl/fl^ mice to generate *S100a8*-Cre/Rosa-Tomato^fl/fl^ mice, where expression of tdTomato is a surrogate marker for *S100a8*-Cre-recombinase activity (**Supplementary Figure 2A**). Flow cytometric analysis demonstrated that over 94% of infiltrating neutrophils in the lungs of *S100a8*-Cre/Rosa-Tomato^fl/fl^ animals were tdTomato^+^ (**Supplementary Figures 1**, **2B, C**). In agreement with previous reports (65), a small fraction of Ly6C^-^ and inflammatory monocytes were also expressing tdTomato at steady state, while no tdTomato expression was observed in alveolar macrophages or lymphocyte populations (**Supplementary Figures 2B, C**). The population of tdTomato^+^ cells of the respiratory tract, as well as the double–positive tdTomato/Ly6G-expressing neutrophils, residing in the red pulp of the spleen, were increased upon IAV infection (**Supplementary Figures 2D, E**).

**Figure 1:**
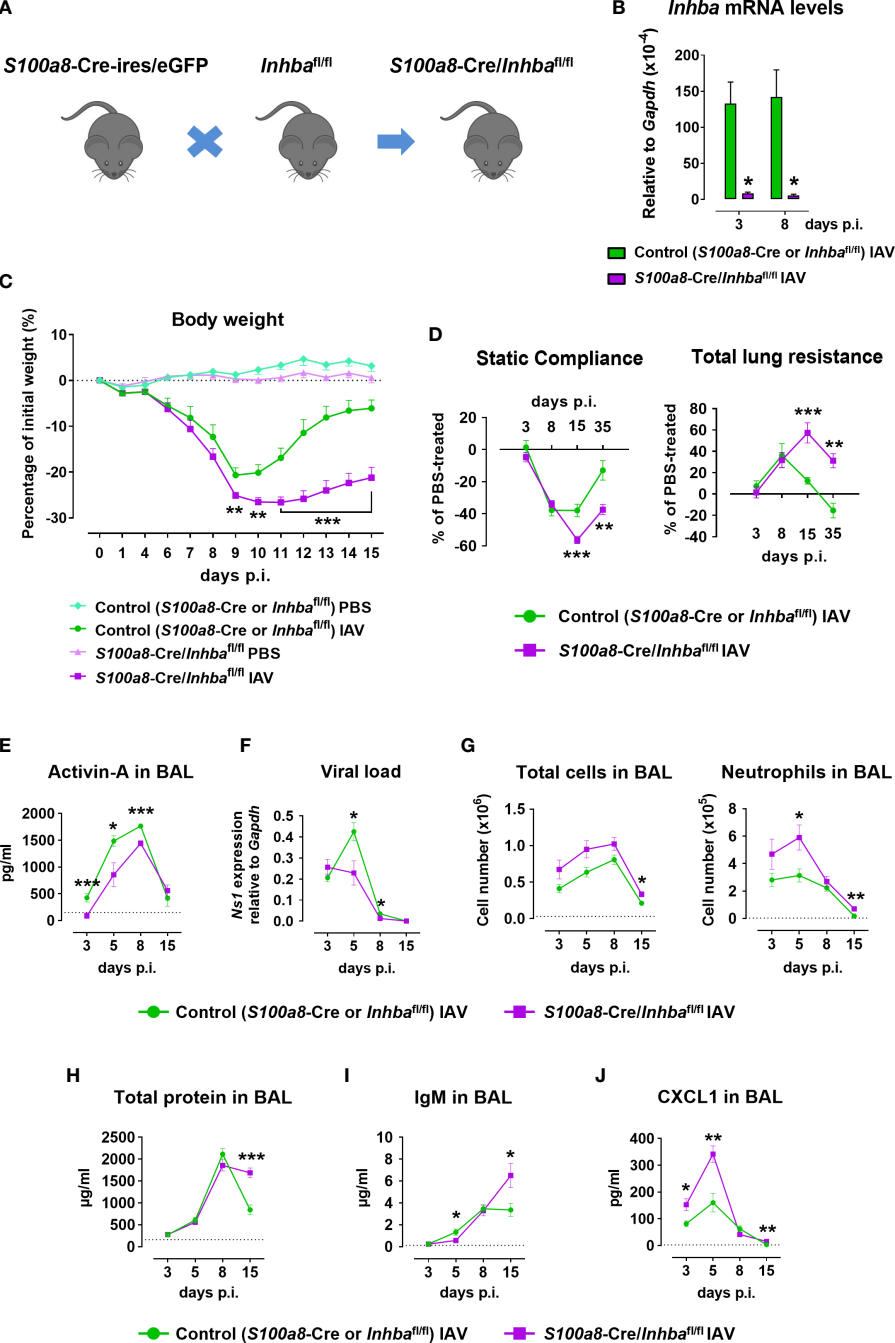
Neutrophil-specific deletion of Activin-A exacerbates pathology in Influenza A virus-infected animals. **(A)** Schematic of experimental animals used in the study. *S100a8*-Cre-ires/eGFP were crossed with *Inhba* ^fl/fl^ to generate *S100a8*-Cre/*Inhba* ^fl/fl^ mice. **(B)** RT-qPCR analysis of mRNA levels for *Inhba* in FACS-sorted neutrophils infiltrating the lungs of IAV-infected control (*S100a8*-Cre or *Inhba* ^fl/fl^) and *S100a8*-Cre/*Inhba* ^fl/fl^ mice, at 3 and 8 days p.i. Data are expressed as mean ± SEM of 3-4 animals/group. **(C)** Body weight expressed as percentage of the initial weight (day 0) of control (*S100a8*-Cre or *Inhba* ^fl/fl^) and *S100a8*-Cre/*Inhba* ^fl/fl^ mice, intranasally inoculated with PBS or 50 pfu of IAV. Data are expressed as mean ± SEM of 11-18 animals/group pooled from two to three independent experiments. Asterisks indicate significant differences compared to control IAV-infected mice. **(D)** Baseline-corrected static compliance and total lung resistance of IAV-infected control and *S100a8*-Cre/*Inhba* ^fl/fl^ mice, at 3, 8, 15, and 35 days p.i. **(E)** Activin-A levels in the BAL of control and *S100a8*-Cre/*Inhba* ^fl/fl^ IAV-treated mice, measured by ELISA. Dotted line represents Activin-A concentration in the BAL of untreated animals (~150 pg/ml) (25). **(F)** Viral load in the lungs of control and *S100a8*-Cre/*Inhba* ^fl/fl^ IAV-treated mice, assessed by RT-qPCR analysis of *Ns1* viral gene expression. **(G)** Numbers of total leukocytes and neutrophils in the BAL of IAV-infected control and *S100a8*-Cre/*Inhba* ^fl/fl^ mice, at 3, 5, 8, and 15 days p.i., as determined by May-Grünwald-Giemsa staining of cytospins. Dotted lines represent the respective cell numbers in the BAL of PBS-treated animals. **(H)** Total protein content in the BAL of control and *S100a8*-Cre/*Inhba* ^fl/fl^ IAV-treated mice as determined by Bradford assay. Dotted line represents the total protein content in the BAL of PBS-treated animals (160.5 ± 10.9 μg/ml). **(I)** IgM levels in the BAL of control and *S100a8*-Cre/*Inhba* ^fl/fl^ IAV-treated mice, measured by ELISA. Dotted line represents IgM concentration in the BAL of PBS-treated animals (121.5 ± 28.4 ng/ml). **(J)** CXCL1 protein levels in the BAL of control and *S100a8*-Cre/*Inhba* ^fl/fl^ IAV-treated mice, analyzed using Milliplex technology. Dotted line represents CXCL1 levels in the BAL of PBS-treated animals (2.4 ± 0.7 pg/ml). Data in **(D–J)** are expressed as mean ± SEM of 5-24 animals/group from two to three independent experiments. Nonparametric Mann-Whitney test was used in all panels, *p < 0.05, **p < 0.01 and ***p < 0.001. BAL, bronchoalveolar lavage fluid; IAV, Influenza A virus; IgM, immunoglobulin M; p.i., post-infection.

To assess the role of neutrophil-derived Activin-A, mice carrying floxed *Inhba* alleles (*Inhba* ^tm3Zuk^) were crossed with the *S100a8*-Cre mice (**Figure 1A**). The consequences of neutrophil-specific Activin-A deficiency were investigated in the context of Influenza A virus-related pathophysiology. *S100a8*-Cre or *Inhba* ^fl/fl^ (hereafter referred to as “control” animals) and *S100a8*-Cre/*Inhba* ^fl/fl^ mice were intranasally infected with 50 pfu of A/PR/8/34 (H1N1) Influenza virus, and *Inhba* expression was assessed in FACS-sorted immune cells infiltrating the lung, 3 and 8 days post-infection (p.i.). Among all cell types, neutrophils had the highest relative expression of the *Inhba* gene (**Figure 1B**; **Supplementary Figure 3A**). Neutrophils infiltrating the lungs of IAV-infected *S100a8*-Cre and *Inhba* ^fl/fl^ parental strains expressed similar levels of *Inhba* mRNA (**Supplementary Figure 4A**). Of interest, its expression was dramatically reduced in neutrophils of *S100a8*-Cre/*Inhba* ^fl/fl^ *versus* control mice at both time points, while its expression was unaltered in other immune or non-immune populations of the lung (**Figure 1B**; **Supplementary Figure 3A**). Some reduction of the *Inhba* mRNA was also observed in Ly6C^-^ and inflammatory monocytes infiltrating the lungs of *S100a8*-Cre/*Inhba* ^fl/fl^ mice; however, this reduction was not statistically significant (**Supplementary Figure 3A**). Moreover, mRNA levels of Follistatin (*Fst*), the natural inhibitor of Activin-A, were increased selectively in non-immune (CD45^-^) cells of the lung following IAV infection (**Supplementary Figure 3B**). Of note, *Fst* mRNA levels were higher in *S100a8*-Cre/*Inhba* ^fl/fl^ CD45^-^ lung cells, compared to the respective control cells, isolated from IAV-infected animals.

To investigate the functional consequences of neutrophil-specific Activin-A deficiency, *S100a8*-Cre/*Inhba* ^fl/fl^ and control animals were inoculated with IAV or PBS (as uninfected control) and monitored up to 15 days p.i. for weight loss. Both IAV-infected groups exhibited similar weight loss up to day 9 p.i. (**Figure 1C**). Interestingly, while control mice infected with IAV began to recover 10 days p.i., mice lacking Activin-A in neutrophils exhibited a delayed recovery of weight loss. Mice inoculated with PBS did not exhibit any weight loss (**Figure 1C**). Moreover, mice were analyzed at specific time points for lung function. Static compliance, reflecting the stiffness of the lung, was significantly reduced in IAV-infected *S100a8*-Cre/*Inhba* ^fl/fl^ mice, 15 and 35 days p.i. (**Figure 1D**). Reversely, total lung resistance, indicative of the resistance of the airflow through the respiratory tract, was significantly increased in IAV-infected *S100a8*-Cre/*Inhba* ^fl/fl^ mice compared to controls, 15 and 35 days p.i. (**Figure 1D**).

Analysis of BAL samples revealed that Activin-A protein was abundantly expressed in the lung 5-8 days after IAV infection (**Figure 1E**). Moreover, Activin-A protein levels were reduced by approximately 20-40% in IAV-infected *S100a8*-Cre/*Inhba* ^fl/fl^ animals in comparison to IAV-infected controls (**Figure 1E**). Activin-A likely produced by cells other than neutrophils may account for the remaining Activin-A expression in IAV-infected *S100a8*-Cre/*Inhba* ^fl/fl^ animals (**Supplementary Figure 3A**). This data suggested that the worsened lung pathology in IAV-infected *S100a8*-Cre/*Inhba* ^fl/fl^ animals was not due to complete absence of Activin-A. The phenotype of *S100a8*-Cre and *Inhba* ^fl/fl^ parental strains in response to IAV was similar in all analyzed parameters (**Supplementary Figures 4B–F**).

Further, analysis of the viral load in the lungs revealed that the exacerbated pathology of *S100a8*-Cre/*Inhba* ^fl/fl^ mice was not due to increased viral proliferation. On the contrary, at 5 and 8 days p.i., control mice had a higher viral titer compared to *S100a8*-Cre/*Inhba* ^fl/fl^ mice (**Figure 1F**). Nevertheless, IAV-infected *S100a8*-Cre/*Inhba* ^fl/fl^ mice exhibited higher inflammation in the BAL compared to control mice (**Figure 1G**), which was mainly due to increased infiltration by neutrophils and macrophages at 5 and 8 days p.i. (**Figure 1G**; **Supplementary Figures 5A, B**). Consistent with the sustained inflammation observed in their lungs, total protein content and IgM levels, as indices of epithelial permeability, were significantly elevated in the BAL of IAV-infected animals carrying Activin-A-deficient neutrophils, compared to the control group, at 15 days p.i. (**Figures 1H, I**).

Next, flow cytometric analysis of the inflammatory cells infiltrating the lungs upon IAV infection showed that mice with Activin-A-deficient neutrophils were characterized by elevated numbers of CD45^+^ leukocytes in comparison to control animals (**Supplementary Figure 5C**). This increase was mainly due to increased infiltration of neutrophils, Ly6C^-^ and inflammatory monocytes, CD4^+^ and CD8^+^ T cells, and B cells. Notably, the absolute number of infiltrating neutrophils remained significantly increased in the lungs of IAV-infected *S100a8*-Cre/*Inhba* ^fl/fl^ animals, even at 15 days p.i. (**Supplementary Figure 5C**). Moreover, increased mRNA expression of inflammatory cytokines and chemokines, namely *Ifnl2/3*, *Il1b*, and *Cxcl1*, was observed in the *S100a8*-Cre/*Inhba* ^fl/fl^ lungs at 3 days p.i. (**Supplementary Figure 5D**). These factors have been recognized as components of feedforward inflammatory circuits during early IAV infection and linked to acute lung injury (55). Of note, increased CXCL1 protein levels were also detected in the BAL of IAV-infected *S100a8*-Cre/*Inhba* ^fl/fl^ animals, at 3, 5, and 15 days p.i. (**Figure 1J**), consistent with the increased recruitment of neutrophils in their lungs (**Figure 1G**; **Supplementary Figure 5C**). Protein levels of additional inflammatory cytokines and chemokines were also analyzed in the BAL of IAV-infected control and *S100a8*-Cre/*Inhba* ^fl/fl^ animals (**Supplementary Figure 6**).

### 3.2 Activin-A deletion from neutrophils leads to increased inflammation and widespread NETosis upon IAV infection

**Figure 2:**
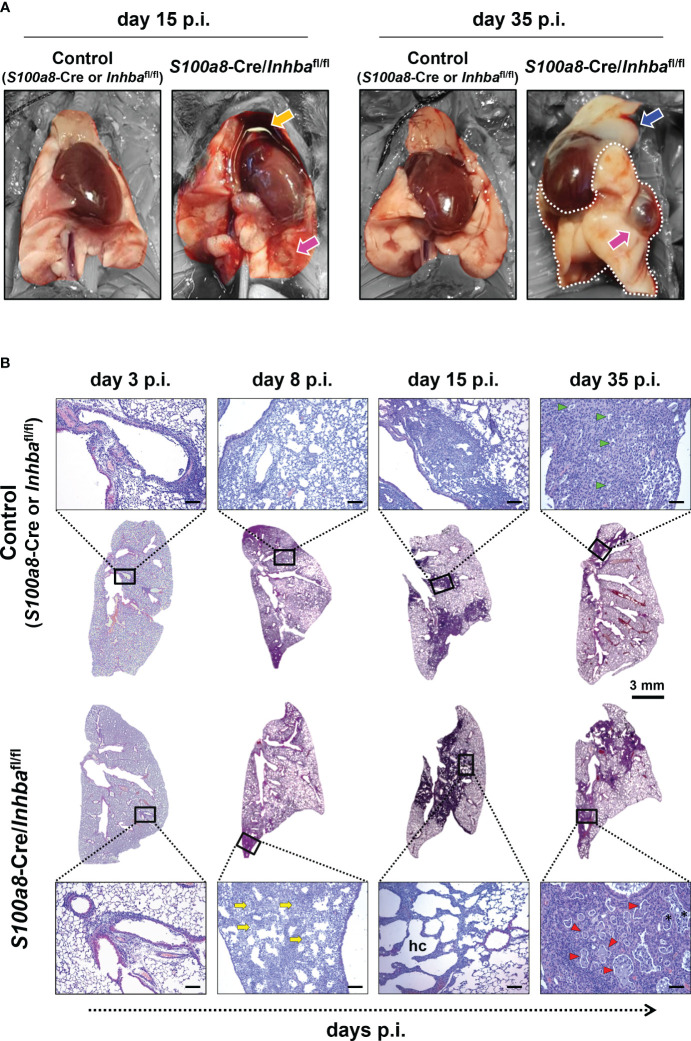
Animals carrying Activin-A-deficient neutrophils are characterized by exacerbated lung histopathology when infected by IAV. **(A)** Representative macroscopic images of the lungs of IAV-infected control (*S100a8*-Cre or *Inhba* ^fl/fl^) and *S100a8*-Cre/*Inhba* ^fl/fl^ mice, at 15 and 35 days p.i. Orange arrow shows the involution of the thymus, blue arrow shows thymus mass restoration, and magenta arrows show focal regions of emphysema (bullae). Parts of the images not corresponding to lung tissue were selectively desaturated for a better presentation of the lungs. **(B)** Representative whole lung sections (Scale bar, 3 mm) and higher magnification images (outlined with a black rectangle; Scale bars, 150 μm) of IAV-infected control (*S100a8*-Cre or *Inhba* ^fl/fl^) and *S100a8*-Cre/*Inhba* ^fl/fl^ mice, stained with H&E, at 3, 8, 15, and 35 days p.i. The selected images represent a view of the typical lesion phenotype, illustrating the “worst-case” phenotype in each group. Yellow arrows show granulocytic foci, green and red arrowheads show small and large proteinaceous foci, respectively, and asterisks show admixed cells. hc, alveolar honeycombing; p.i., post-infection.

Macroscopic assessment of lung pathology 15 days p.i. revealed that neutrophil-specific deletion of Activin-A was associated with widespread hemorrhagic areas in the lungs and severe involution of the thymus, compared to the control group (**Figure 2A**). While thymic tissue mass was later restored, the lungs of IAV-infected *S100a8*-Cre/*Inhba* ^fl/fl^ animals were characterized by persistent tissue remodeling with focal regions of emphysema-bullae, at 35 days p.i. (**Figure 2A**).

**Figure 3:**
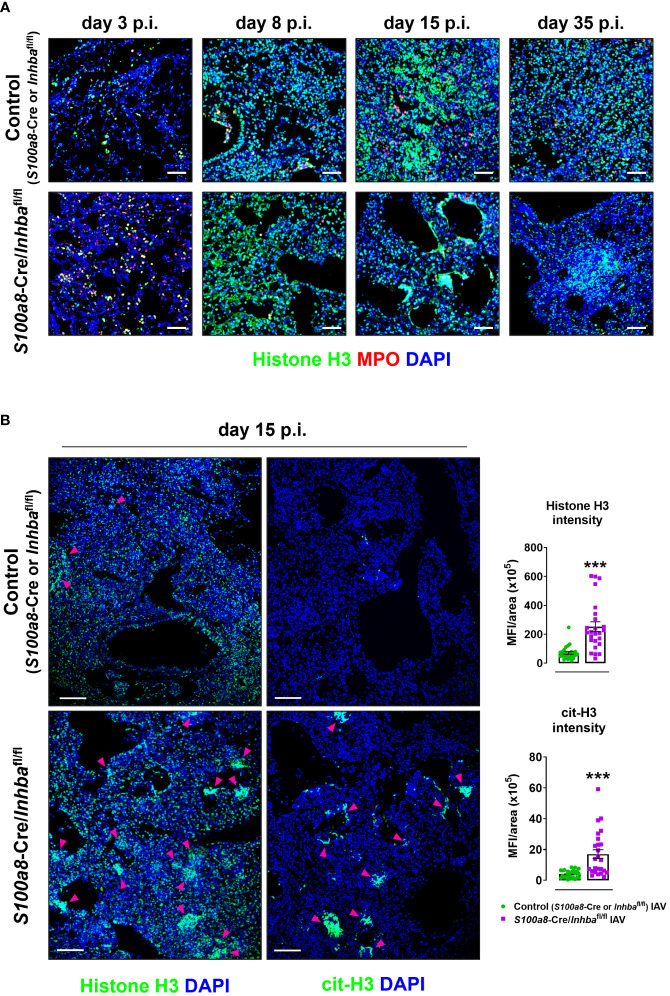
Hallmark of the exacerbated lung histopathology observed in *S100a8*-Cre/*Inhba* ^fl/fl^ IAV-infected mice is widespread NETosis. **(A)** Representative confocal immunofluorescence images for histone H3 (green) and MPO (red) in lung sections of control (*S100a8*-Cre or *Inhba* ^fl/fl^) and *S100a8*-Cre/*Inhba* ^fl/fl^ IAV-infected mice, at 3, 8, 15, and 35 days p.i. Scale bars, 50 μm. **(B)** Representative confocal immunofluorescence images for histone H3 or cit-H3 (green) in lung sections of IAV-infected control (*S100a8*-Cre or *Inhba* ^fl/fl^) and *S100a8*-Cre/*Inhba* ^fl/fl^ mice, 15 days p.i. Scale bars, 100 μm. Arrowheads indicate clusters of NETotic cells. DAPI (blue) was used for nuclear staining. Graphs depict the Fiji/ImageJ-assisted quantification of MFI of histone H3 and cit-H3, normalized over the area. Data are expressed as mean ± SEM of 25 fields/group. Nonparametric Mann-Whitney test was used, ***p < 0.001. cit-H3, citrullinated histone H3; IAV, Influenza A virus; MFI, mean fluorescence intensity; MPO, myeloperoxidase; p.i., post-infection.

To assess the extent of inflammation at the tissue level, we performed H&E staining of lung sections, 3, 8, 15, and 35 days p.i. Consistent with our macroscopic observations, H&E-stained sections revealed severe tissue injury and gradual development of honeycomb structures in *S100a8*-Cre/*Inhba* ^fl/fl^ mice, compared to the respective controls, following IAV infection (**Figure 2B**; **Supplementary Figure 7**). More specifically, IAV-infected control animals showed multifocal perivascular and peribronchiolar inflammatory cell infiltrates at 3 days p.i., maturing to diffuse pneumonitis by day 8 p.i., which showed multifocal consolidation by day 15 p.i. (**Figure 2B**). By comparison, the lungs of IAV-infected *S100a8*-Cre/*Inhba* ^fl/fl^ animals showed a similar histological phenotype to controls at 3 days p.i.; however, they were characterized by a transition to a more severe histological phenotype from 8 days p.i. and onwards; a transition from diffuse pneumonitis with prominent granulocytic foci at 8 days p.i. to a marked multifocal alveolar honeycombing at 15 days p.i. (**Figure 2B**). At this time point, lung sections stained with DAPI revealed a widespread deposition of extranuclear and extracellular chromatin within the injured tissues of IAV-infected *S100a8*-Cre/*Inhba* ^fl/fl^ animals (**Supplementary Figure 8A**). This observation led us to consider the involvement of NET formation since previous studies have reported that upon IAV infection neutrophils undergo NETosis that contributes to acute lung damage and exacerbates tissue injury (15, 66). Indeed, immunofluorescence staining of lung sections using antibodies against histone H3 and MPO revealed the abundant presence of NETs in the lungs of IAV-infected *S100a8*-Cre/*Inhba* ^fl/fl^ mice, compared to control animals, peaking at 15 days p.i. (**Figures 3A, B**; **Supplementary Figure 8B**). Interestingly, NETs were observed in areas of extended pathology, lining alveolar regions with a honeycombing appearance. These areas were also positive for citrullinated histone H3 (cit-H3), further strengthening the hypothesis that the extracellular chromatin, observed in the lungs of *S100a8*-Cre/*Inhba* ^fl/fl^ mice upon IAV infection, was due to extensive NET formation (**Figure 3B**; **Supplementary Figure 8B**).

Eventually, at 35 days p.i., fewer consolidated lesions were detected in IAV-infected control mice than on day 15, with apparent small, focal, proteinaceous areas, admixed with cell debris (**Figure 2B**). On the other hand, by day 35, *S100a8*-Cre/*Inhba* ^fl/fl^ lungs showed less honeycombing, replaced by a prominent confluent pneumonic consolidation with large proteinaceous fluid-filled cystic areas, admixed with cell debris, and surrounded by atypical epithelial cells (**Figure 2B**). Overall, Activin-A deletion from *S100a8*-expressing cells disturbed lung homeostasis upon IAV infection, leading to increased inflammation, widespread NETosis, and persisting tissue damage.

### 3.3 Neutrophils are potential targets of a self-regulating feedback loop of their own secreted Activin-A

**Figure 4:**
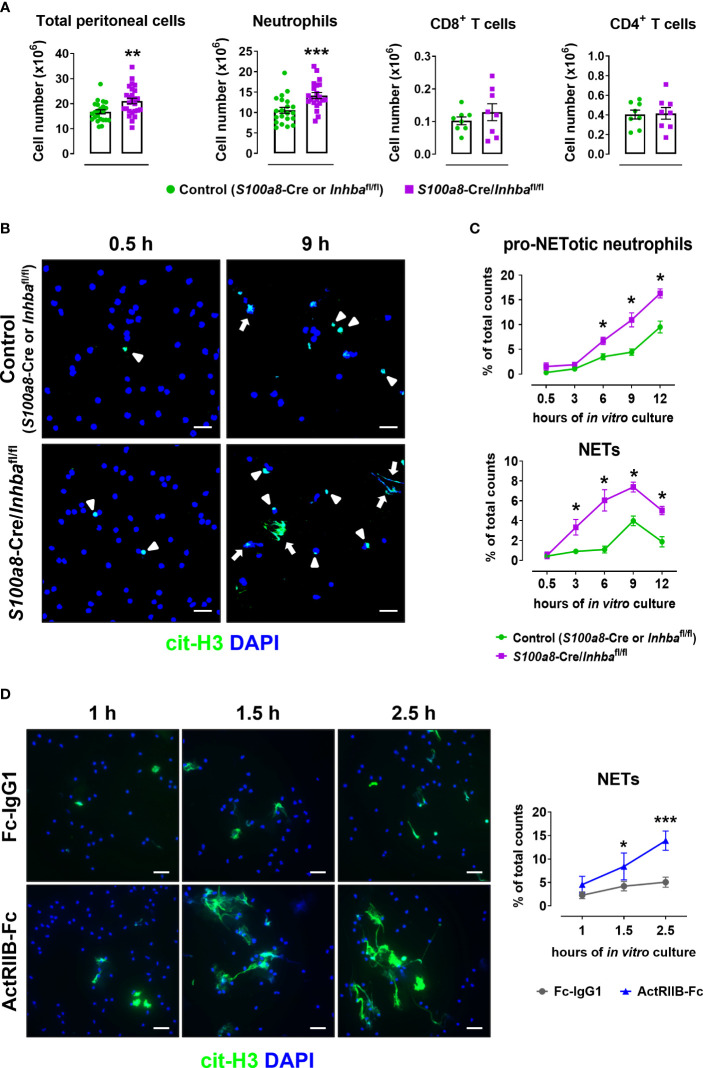
Activin-A secreted by neutrophils moderates their predisposal for NET release. **(A)** Numbers of total peritoneal cells, neutrophils, CD8^+^ T cells, and CD4^+^ T cells, as analyzed by flow cytometry in control (*S100a8*-Cre or *Inhba* ^fl/fl^) and *S100a8*-Cre/*Inhba* ^fl/fl^ thioglycollate (TG)-injected mice, six hours post-injection. Data are expressed as mean ± SEM of 21-26 animals/group for total peritoneal cells and neutrophils, and eight animals/group for T cells, from three independent experiments. **(B)** Representative confocal microscopy images for cit-H3 (green) in neutrophils isolated from control (*S100a8*-Cre or *Inhba* ^fl/fl^) and *S100a8*-Cre/*Inhba* ^fl/fl^ TG-injected mice, at 0.5 and 9 hours of *in vitro* culture. Arrowheads indicate pro-NETotic neutrophils (cit-H3/DAPI double-positive intact cells), and arrows indicate NETs (extranuclear and extracellular cit-H3^+^ chromatin deposition). Scale bars, 25 μm. **(C)** Quantitation of cit-H3^+^ cells by Fiji/ImageJ-assisted analysis expressed as a percentage of pro-NETotic neutrophils and NETs in total counts. Neutrophils were isolated from control (*S100a8*-Cre or *Inhba* ^fl/fl^) and *S100a8*-Cre/*Inhba* ^fl/fl^ TG-injected mice and cultured for 0.5, 3, 6, 9, and 12 hours *in vitro*. Data are expressed as mean ± SEM of four animals/group. **(D)** Representative confocal microscopy images for cit-H3 (green) in human peripheral blood neutrophils, cultured in the presence of 10 μg/ml ActRIIB-Fc or control human IgG1-Fc for 1, 1.5, and 2.5 hours. Graph depicts the Fiji/ImageJ-assisted analysis of *in vitro* NETosis, expressed as a percentage of cit-H3^+^ cells in total counts. Neutrophils were isolated from four different healthy donors. Data are expressed as mean ± SEM of 6-10 samples/group, from two independent experiments. Scale bars, 25 μm. DAPI (blue) was used for nuclear staining. For the experiments presented in panels **(B–D)**, 1.5x10^5^ neutrophils per well were plated (on PDL-coated coverslips) in a 24-well plate. Nonparametric Mann-Whitney test was used in all panels, *p < 0.05, **p < 0.01, and ***p < 0.001. ActRIIB-Fc, fusion of the extracellular domain of the Activin type IIB receptor to the Fc fragment of human IgG1; cit-H3, citrullinated histone H3; NETs, neutrophil extracellular traps.

To confirm the inherent predisposal of Activin-A-deficient neutrophils for NET release, *S100a8*-Cre/*Inhba* ^fl/fl^ and control mice were intraperitoneally injected with 4% thioglycollate, and peritoneal cells were collected six hours post-injection when the peak of neutrophilia is observed. Neutrophils (CD45^+^CD11b^+^Ly6G^+^ cells) represented 0.7 ± 0.1% and 65.2 ± 1.2% of live CD45^+^ peritoneal cells in PBS-injected and thioglycollate-injected animals, respectively, as analyzed by flow cytometry six hours post-injection (**Supplementary Figure 9**). The total cell number in the peritoneal cavity of *S100a8*-Cre/*Inhba* ^fl/fl^ mice was significantly higher compared to control mice, mainly because of an increased neutrophil influx, as revealed by flow cytometric analysis (**Figure 4A**). Interestingly, peritoneal neutrophils from *S100a8*-Cre/*Inhba* ^fl/fl^ mice were more prone to spontaneously form cit-H3^+^ NETs, when cultured *in vitro*, compared to control neutrophils (**Figures 4B, C**). However, no changes were observed between control and Activin-A-deficient peritoneal neutrophils in mitochondrial membrane polarization, reactive oxygen species, or nitric oxide production, suggesting cells to be both equally metabolically active, at least after thioglycollate injection (**Supplementary Figure 10**).

Since deletion of Activin-A from murine neutrophils enhanced both *in vivo* and *in vitro* their pro-NETotic tendency, we investigated whether neutralizing Activin-A secreted by normal human neutrophils in culture could also impact their activation towards NET formation. To this end, peripheral blood neutrophils from healthy individuals were cultured in the presence of ActRIIB-Fc, a fusion protein that binds and neutralizes Activin-A (25), or human IgG1-Fc as control. Interestingly, treatment of human neutrophils with ActRIIB-Fc significantly enhanced the spontaneous release of cit-H3^+^ NETs, compared to the control cultures, at 1.5 and 2.5 hours of *in vitro* culture (**Figure 4D**), suggesting that at least part of the neutrophil-derived Activin-A effect is mediated through direct modulation of neutrophil function.

**Figure 5:**
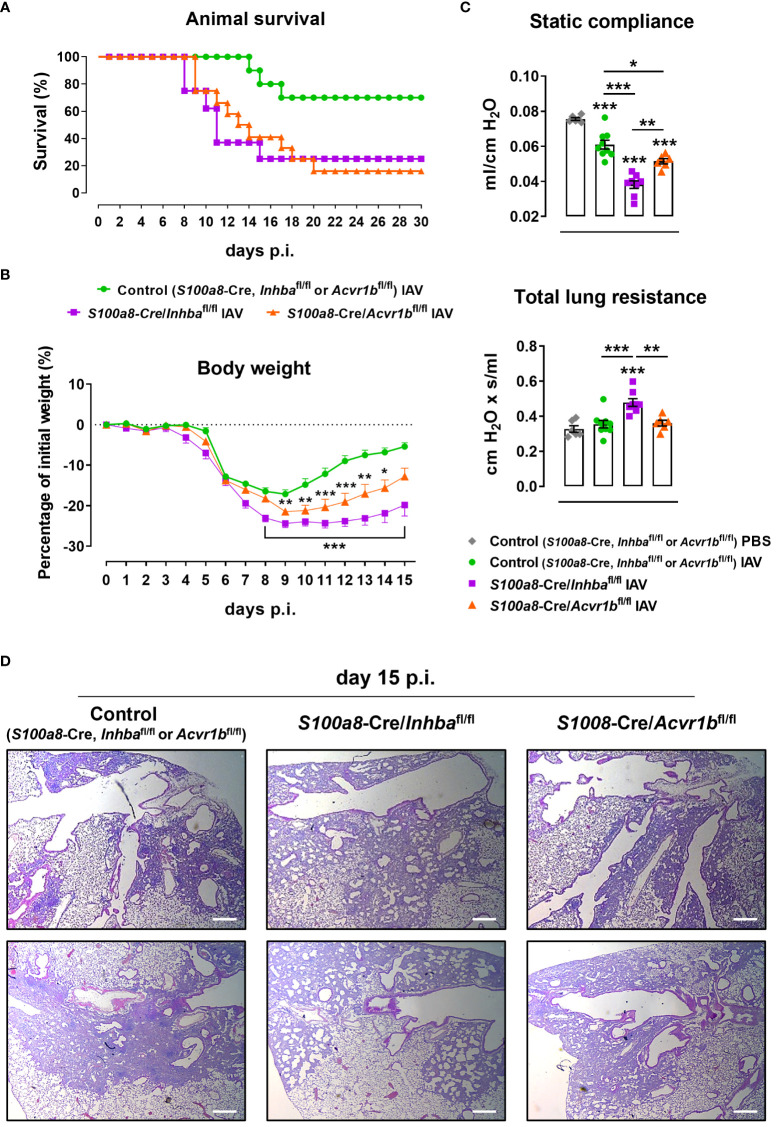
ALK4-deficiency in neutrophils enhances sensitivity to IAV infection. **(A)** Survival curve of control (*S100a8*-Cre, *Inhba* ^fl/fl^ or *Acvr1b* ^fl/fl^), *S100a8*-Cre/*Inhba* ^fl/fl^, and *S100a8*-Cre/*Acvr1b* ^fl/fl^ mice intranasally inoculated with 500 pfu of IAV (10-12 animals/group). **(B)** Body weight expressed as percentage of the initial weight (day 0) of control, *S100a8*-Cre/*Inhba* ^fl/fl^, and *S100a8*-Cre/*Acvr1b* ^fl/fl^ mice, inoculated with 50 pfu of IAV. Data are expressed as mean ± SEM of 6-8 animals/group. Asterisks indicate significant differences from the control IAV group. **(C)** Static compliance and total lung resistance of control, *S100a8*-Cre/*Inhba* ^fl/fl^, and *S100a8*-Cre/*Acvr1b* ^fl/fl^ mice, intranasally inoculated with PBS or 50 pfu of IAV, 15 days p.i. Data are expressed as mean ± SEM of 6-9 animals/group. Asterisks without horizontal lines indicate significant differences from the control PBS group, while asterisks with horizontal lines represent the comparison between the groups under the line. One-way ANOVA, followed by Bonferroni’s *post hoc* test, was used in all panels, *p < 0.05, **p < 0.01, and ***p < 0.001. **(D)** Representative lung sections of control, *S100a8*-Cre/*Inhba* ^fl/fl^, and *S100a8*-Cre/*Acvr1b* ^fl/fl^ IAV-infected mice stained with H&E, at 15 days p.i. Scale bars, 300 μm. IAV, Influenza A virus; p.i., post-infection.

To further assess whether neutrophils are direct targets of the Activin-A they secrete, we developed animals with neutrophils deficient for the Activin-A type I receptor ALK4/ACVR1B (*S100a8*-Cre/*Acvr1b* ^fl/fl^ mice). Administration of a lethal dose of IAV (500 pfu) led to increased mortality of both Activin-A- and ALK4/ACVR1B-deficient groups in comparison to control (*S100a8*-Cre, *Inhba* ^fl/fl^ or *Acvr1b* ^fl/fl^) mice, starting from day 8 p.i. (**Figure 5A**). Of note, upon IAV infection, only 16% of *S100a8*-Cre/*Acvr1b* ^fl/fl^ and 25% of *S100a8*-Cre/*Inhba* ^fl/fl^ mice survived after day 20 p.i., in contrast to the 75% survival observed in the control group (**Figure 5A**).

Following infection with a non-lethal dose of IAV (50 pfu), *S100a8*-Cre/*Acvr1b* ^fl/fl^ animals exhibited a worse phenotype compared to controls (**Figures 5B–D**). However, in comparison to *S100a8*-Cre/*Inhba* ^fl/fl^ animals, they exhibited intermediate levels of weight loss (**Figure 5B**) and static compliance decrease (**Figure 5C**). Similarly, H&E-stained lung sections of IAV-infected *S100a8*-Cre/*Acvr1b* ^fl/fl^ animals revealed a moderate degree of tissue injury with a more prominent pneumonitis compared to controls, which did not involve the extreme honeycomb appearance of *S100a8*-Cre/*Inhba* ^fl/fl^ lungs (**Figure 5D**).

During neutrophil development, the *Acvr1b* gene is activated transcriptionally earlier than the *Inhba* and *S100a8* genes, which are activated with similar kinetics (**Supplementary Figure 11A**, (43)). Therefore, it is possible that due to the relative delay in the expression of Cre recombinase in the *S100a8*-Cre/*Acvr1b* ^fl/fl^ animals, the developing neutrophils have time to synthesize some *Acvr1b* mRNA before the gene is deleted, leading to the milder phenotype described above (**Figures 5B–D**). In line with this reasoning, *Acvr1b* mRNA expression in bone marrow cells showed a ~50% reduction in *S100a8*-Cre/*Acvr1b* ^fl/fl^ and *S100a8*-Cre/*Inhba* ^fl/fl^ animals, whereas *Inhba* expression was almost eliminated in the bone marrow of *S100a8*-Cre/*Inhba* ^fl/fl^ mice (**Supplementary Figure 11B**).

### 3.4 Neutrophil-specific Activin-A deficiency is associated with transcriptome alterations consistent with a predisposal for NET release

**Figure 6:**
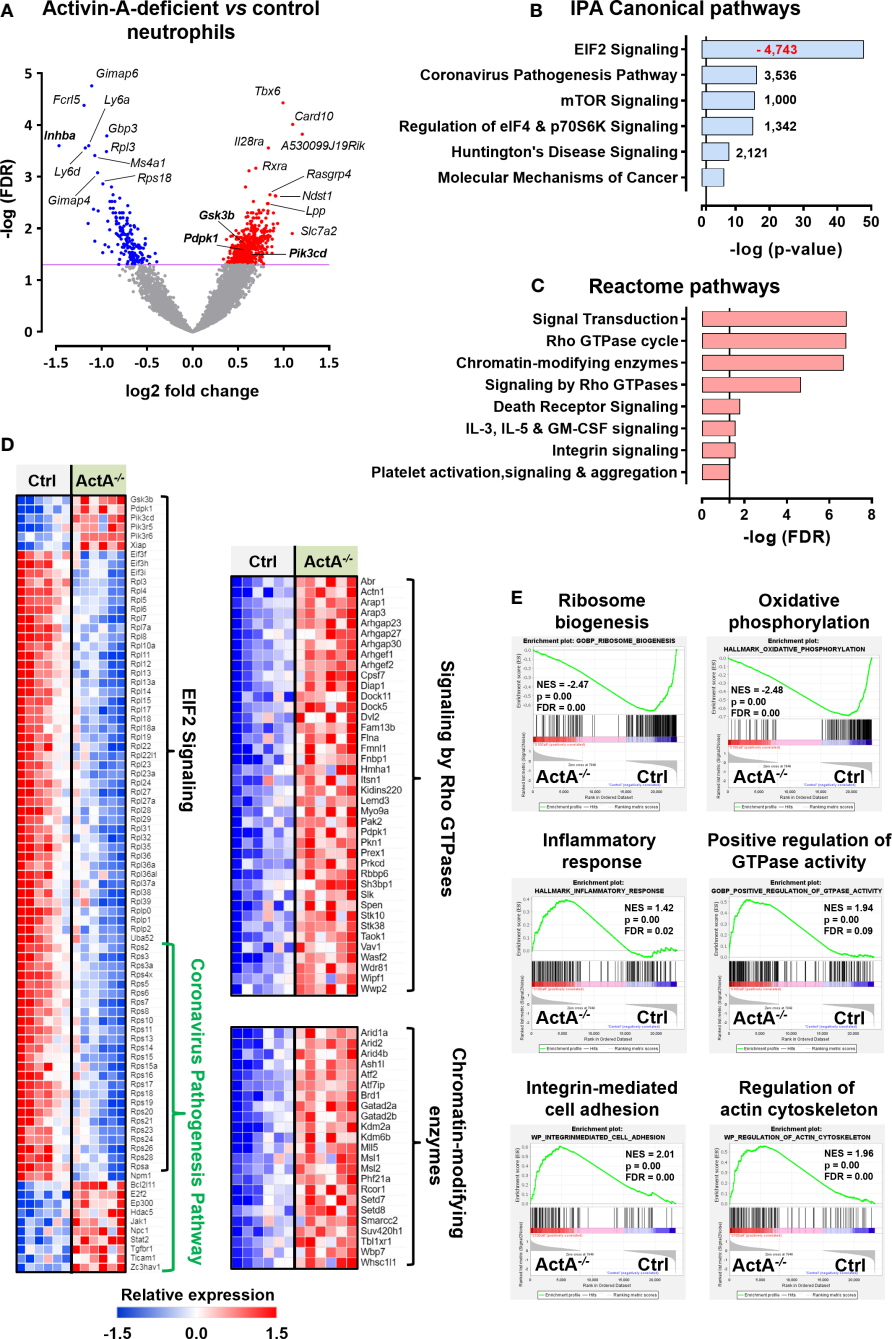
Transcriptome analysis of control and Activin-A*-*deficient neutrophils. **(A)** Volcano plot depicting the differentially expressed genes (DEGs) in *S100a8*-Cre/*Inhba* ^fl/fl^ *versus* control (*S100a8*-Cre or *Inhba* ^fl/fl^) neutrophils, following RNA-Seq analysis. Neutrophils were purified by cell sorting from the peritoneum of thioglycollate-injected animals, six hours post-injection (6 samples/group). **(B)** Graph depicting the top regulated IPA canonical pathways from RNA-Seq analysis of *S100a8*-Cre/*Inhba* ^fl/fl^ *versus* control neutrophils. Numbers next to the bars represent IPA z-scores predicting increased (black numbers) or decreased (red numbers) pathway activity. **(C)** Graph depicting the top regulated Reactome pathways of upregulated DEGs, derived from RNA-Seq analysis of *S100a8*-Cre/*Inhba* ^fl/fl^ *versus* control neutrophils. Pathways with redundant DEGs were excluded. Vertical lines in **(B, C)** show the threshold for statistical significance (p-value or FDR < 0.05). **(D)** Heatmaps depicting the relative expression of DEGs belonging to the top IPA or Reactome pathways, as determined by RNA-Seq analysis of purified neutrophils. **(E)** Gene set enrichment analysis (GSEA) plots, revealing significantly altered signatures in the transcriptome of purified *S100a8*-Cre/*Inhba* ^fl/fl^ *versus* control neutrophils, using Hallmark, Gene Ontology Biological Process, and Wikipathways as reference gene sets from the Mouse Molecular Signatures Database. ActA^-/-^, *S100a8*-Cre/*Inhba* ^fl/fl^; Ctrl, control (*S100a8*-Cre or *Inhba* ^fl/fl^); EIF2, eukaryotic initiation factor 2; FDR, false discovery rate; IPA, Ingenuity Pathway Analysis; NES, normalized enrichment score.

To provide some mechanistic explanation for the increased pro-NETotic tendency of Activin-A-deficient neutrophils, we compared their transcriptome to control (*S100a8*-Cre or *Inhba* ^fl/fl^) neutrophils. To this end, control and *S100a8*-Cre/*Inhba* ^fl/fl^ neutrophils were collected from the peritoneum of thioglycollate-injected animals, purified by cell sorting, and their transcriptome was analyzed by RNA-Sequencing (RNA-Seq). Six hundred and fourteen differentially expressed genes (DEGs) were identified, of which 440 were up- and 174 were down-regulated in Activin-A-deficient relative to control neutrophils (**Figure 6A**). The expression levels of representative DEGs were further verified by RT-qPCR analysis of independently purified peritoneal neutrophils (**Supplementary Figure 12A**).

Bioinformatics analysis using the Ingenuity Pathway Analysis (IPA) platform highlighted the overrepresentation of translation-, inflammation-, and cell metabolism-related canonical pathways, including “EIF2 signaling”, “Coronavirus pathogenesis pathway”, “mTOR signaling”, and “Regulation of eIF4 and p70S6K signaling” (**Figure 6B**). The top-regulated IPA pathway was “EIF2 signaling”, which was characterized by a negative IPA z-score (predictive of decreased activity), as several genes encoding for structural proteins of both small and large ribosome subunits or regulators of protein synthesis were significantly downregulated in *S100a8*-Cre/*Inhba* ^fl/fl^ neutrophils (**Figures 6B, D**). Focused analysis on the upregulated DEGs only, using the GeneCodis4 web-based tool, unraveled several signaling transduction pathways of the Reactome database (**Figure 6C**). Taken together, decreased expression of genes encoding for ribosomal proteins or regulators of protein synthesis and increased expression of inflammatory mediators, Rho GTPase cycle components, and chromatin-modifying enzymes characterized the transcriptome of Activin-A-deficient neutrophils (**Figure 6D**). Analysis of all the DEGs belonging to the top IPA and Reactome pathways using the STRING database illustrated a potential functional connection between the up- and down-regulated clusters of genes and, interestingly, placed PI3K/AKT signaling components at the interface of these top-regulated pathways (**Supplementary Figure 12B**). Activation of PI3K/AKT signaling in neutrophils has been closely associated with NET formation (67–69), implicating autophagy in this process (70, 71).

Gene set enrichment analysis (GSEA) was also performed to reveal enriched signatures in the dataset, independently verifying the downregulation of “ribosome biogenesis” in *S100a8*-Cre/*Inhba* ^fl/fl^ neutrophils (**Figure 6E**). GSEA analysis also highlighted the increased “inflammatory response”, “integrin-mediated cell adhesion”, “regulation of actin cytoskeleton”, “positive regulation of GTPase activity”, and altered “oxidative phosphorylation” for the Activin-A-deficient neutrophils at the transcriptional level (**Figure 6E**). Overall, the downregulation of “ribosome biogenesis” and translation-related pathways, together with the increased expression of “chromatin modification” and “regulation of actin cytoskeleton” components, were consistent with the increased pro-NETotic tendency of Activin-A-deficient neutrophils (72–74).

## 4 Discussion

Neutrophils, armed with potent anti-microbial mechanisms, represent the first line of host defense that is mobilized in response to any disruption of homeostasis (5–7). Despite their crucial protective role, if left uncontrolled, neutrophils may inflict widespread collateral damage and cause pathology (13, 15). This study provides evidence suggesting that neutrophil-secreted Activin-A, through regulation of their pro-NETotic tendency, acts as a key moderator of their damaging potential.

Activin-A is synthesized at the early stages of neutrophil maturation in the bone marrow (43), to be released at the earliest stage of their activation, following an inflammatory insult in the periphery (44–47). The actual function of Activin-A which is either pre-loaded in neutrophils during their early development or produced *de novo* during their activation in the periphery has not been resolved so far.

Therefore, to clarify the role of neutrophil-derived Activin-A, we selectively deleted it from neutrophils using the *S100a8*-Cre/*Inhba* ^fl/fl^ transgenic system and used the Influenza A virus model to probe neutrophil functionality. IAV-infected animals carrying Activin-A-deficient neutrophils exhibited an enhanced pathology in comparison to IAV-infected control animals, as exemplified by persistent weight loss, altered lung mechanics, increased infiltration of inflammatory cells, increased levels of pro-inflammatory mediators, total protein, and IgM in their BAL, and widespread hemorrhagic lung histopathology. Increased release of potent neutrophil chemoattractants, such as CXCL1, may account for the enhanced recruitment of neutrophils in the lungs of IAV-infected *S100a8*-Cre/*Inhba* ^fl/fl^ animals compared to the control group. The histological hallmark of the pathology observed in IAV-infected animals carrying Activin-A-deficient neutrophils was the exuberant and prolonged NETosis in their lungs, which was associated with severe long-lasting tissue remodeling. The mice eventually recovered, most likely due to the capacity of murine lungs to regenerate throughout life (75, 76). However, in humans, whose lungs do not have similar lifelong regenerative capacity and where prolonged presence of NETs in IAV-infected lung areas can lead to severe pathology (15) and enhanced tissue damage (13, 15, 77, 78), potential mechanisms that could enhance the pro-NETotic tendency of neutrophils are particularly relevant.

The exacerbated phenotype of IAV-infected animals carrying Activin-A-deficient neutrophils does not seem to be due to a complete absence of Activin-A in the airspaces, since their BAL contained approximately 50-70% of the Activin-A levels found in control animals. This Activin-A could derive from other cells in the lung, such as monocytes, macrophages, or epithelial cells that have also been reported to secrete it (34–37). The phenotype observed due to the deficiency of neutrophils in the context of an environment that is not completely devoid of Activin-A raises the possibility that, in addition to its absolute levels in the inflammatory microenvironment, the cellular source of Activin-A is a crucial parameter for its functionality. The cellular source of a cytokine or chemokine could affect the kinetics and/or the precise location of its production. Different types of cells could produce Activin-A at earlier or later stages of the inflammatory response in the bloodstream, the airspaces, or the parenchymal microenvironment. Moreover, the introduction in different cell types of different biochemical modifications that could affect Activin-A functionality, such as glycosylation, cannot be excluded. Interestingly, and along the lines of the above reasoning, selective deletion of Activin-A from mesenchymal cells was associated with reduced tissue injury in an experimental model of arthritis (79), highlighting the importance of the cell origin and context dependency of the Activin-A functionality.

The pro-NETotic tendency of Activin-A-deficient neutrophils was also evident in neutrophils isolated using the thioglycollate-induced peritonitis model. Increased neutrophil influx in the peritoneal cavity and enhanced predisposal for spontaneous *in vitro* NETosis were observed in *S100a8*-Cre/*Inhba* ^fl/fl^ animals and neutrophils, respectively. Interestingly, neutralization of Activin-A in cultures of purified human neutrophils enhanced their spontaneous NETosis, further supporting the notion that the prepacked Activin-A they carry and/or the one they synthesize upon activation targets the neutrophils that secrete it and moderates their activation towards NET formation.

The latter notion was further supported by selectively deleting the Activin-A type I receptor ALK4/ACVR1B from neutrophils. Given the fact that *Acvr1b* mRNA starts being synthesized in the developing neutrophils before the *S100a8* promoter gets activated (43), a knock-down rather than a knock-out of ALK4 expression is anticipated via *S100a8*-Cre-mediated *Acvr1b* ^fl/fl^ deletion, at least in early neutrophils. Indeed, approximately a 50% reduction in *Acvr1b* mRNA expression was observed in bone marrow cells from *S100a8*-Cre/*Acvr1b* ^fl/fl^ animals. Still, after exposure to a lethal dose of IAV in the airways, survival of animals with either Activin-A- or ALK4-deficient neutrophils was equally reduced, while at lower doses of viral exposure, animals with ALK4-deficient neutrophils exhibited a moderate phenotype between the control animals and those carrying Activin-A-deficient neutrophils. These results are consistent with the notion that neutrophils themselves are among the key cellular targets of the Activin-A they carry or produce.

Of course, it is possible that neutrophil-derived Activin-A could moderate collateral tissue damage by acting on other cells in the tissue microenvironment. For instance, Activin-A could moderate the recruitment of inflammatory monocytes in the lung, consistent with the observed increased levels of inflammatory monocytes in IAV-infected *S100a8*-Cre/*Inhba* ^fl/fl^ animals. Activin-A might also induce regulatory T cells, which in turn could suppress inflammatory manifestations in the lung, as has been previously shown in a humanized model of allergic airway inflammation (80). Alternatively, neutrophil-derived Activin-A could affect resident lung cells as well, including fibroblasts, endothelial, mesenchymal, or epithelial cells (81).

Transcriptome analysis of control and Activin-A-deficient peritoneal neutrophils, isolated from thioglycollate-treated animals, was used to provide a putative mechanistic explanation for the pro-NETotic tendency of the latter. More than 600 genes were found differentially up- or down-regulated in the Activin-A-deficient neutrophils, the majority of which could be organized into three main clusters, following analysis with the IPA software and the GeneCodis4 web-based tool. The major cluster consisted of numerous downregulated genes encoding ribosomal proteins and regulators of their biogenesis. The other two clusters, that involved upregulated DEGs, included chromatin-modifying enzymes and components of Rho GTPase signaling. An inverse relationship between protein synthesis and autophagy, the key driver of NETosis, has been repeatedly reported in the literature (72, 82–85). Thus, the downregulation of pathways associated with translation observed in murine Activin-A-deficient neutrophils is consistent with their activation towards NET formation.

Interestingly, following subsequent analysis using the STRING database, components of PI3K/AKT signaling were found to be at the interface of the aforementioned three clusters of DEGs, providing a potential functional connection between them. Indeed, activation of the PI3K/AKT signaling system in neutrophils has been shown to induce NETosis in response to various stimuli (67–69), probably through regulation of autophagy and ROS generation (67, 70, 86). Moreover, a genome-wide transcriptional firing regulated by specific kinase cascades, including mitogen-activated protein (MAP) and AKT kinases, has been reported to be necessary for chromatin decondensation and subsequent NET formation (83).

Deregulation of the Activin-A/Follistatin system has been previously reported for critically ill patients with H1N1 infection, since they were characterized by elevated serum levels of Activin-A, Activin-B, and Follistatin, the natural inhibitor of Activins, at the time of admission to the intensive care unit (27). Increased plasma levels of Follistatin have been associated with in-hospital mortality of COVID-19 patients as well (87) and were shown to be predictive of the fatal outcome at any time of the disease progression (28). Interestingly, we detected increased mRNA expression of Follistatin selectively in CD45^-^ lung cells during IAV infection in mice, with the highest values observed in cells isolated from *S100a8*-Cre/*Inhba* ^fl/fl^ animals. The above may indicate that inhibition of Activin-A from neutrophils or overexpression of Follistatin in the serum or lungs could shift the balance towards a more aggressive, NETosis-prone neutrophil response and the development of NET-associated pathology.

Moreover, mRNA expression of key components of the Activin-A/Follistatin system was significantly increased following IAV infection of human cells *in vitro* (88), independently verifying the interplay between the Activin-A/Follistatin system and IAV-mediated pathology. There is also evidence from integrated single-cell RNA-Seq data to genome-wide association analysis that neutrophils are significantly associated with IAV and COVID-19 infections in a European cohort (89). Besides, severe Influenza infection has been associated with the overrepresentation of neutrophil-related processes in humans, including pathways involved in neutrophil differentiation, migration, degranulation, and NET formation (90), consistent with the NETosis-related fingerprint of murine Activin-A-deficient neutrophils.

Collectively, our findings unveil a novel aspect of the Activin-A/neutrophil liaison, suggesting that Activin-A, apparently synthesized and loaded in neutrophils at an early stage in their development, constitutes a feedback moderator of neutrophil-mediated and NETosis-driven collateral tissue damage.

## Data availability statement

The datasets presented in this study can be found in the NCBI's Sequence Read Archive (SRA) repository. The URL of the repository and accession number can be found below: https://www.ncbi.nlm.nih.gov/sra, PRJNA966106.

## Ethics statement

The studies involving humans were approved by the Scientific and Ethics Committee of the University Hospital of Alexandroupolis, Greece (Approval No. 803/23-09-2019). The participants provided their written informed consent to participate in this study. The animal studies were approved by the Institutional Ethics Committee for Use of Laboratory Animals (BRFAA, Athens, Greece) and the Greek Ministry of Agriculture (Approval No. 2082/07-05-2018). All studies were conducted in accordance with local legislation and institutional requirements.

## Author contributions

GD: Conceptualization, Data curation, Formal analysis, Investigation, Methodology, Software, Validation, Visualization, Writing – original draft, Writing – review & editing. ES: Conceptualization, Data curation, Formal analysis, Investigation, Methodology, Validation, Visualization, Writing – original draft, Writing – review & editing. AD: Investigation, Writing – review & editing, Methodology. AG: Investigation, Methodology, Software, Writing – review & editing, Data curation. CG: Formal analysis, Investigation, Methodology, Writing – review & editing, Data curation. AA: Investigation, Methodology, Software, Writing – review & editing, Data curation. MF: Data curation, Writing – review & editing, Formal analysis. MM: Funding acquisition, Resources, Writing – review & editing. PSk: Funding acquisition, Resources, Writing – review & editing. IG: Data curation, Formal analysis, Writing – original draft, Writing – review & editing, Investigation, Methodology. PSi: Conceptualization, Data curation, Formal analysis, Funding acquisition, Project administration, Supervision, Visualization, Writing – original draft, Writing – review & editing.
