# Supplementary material for: Neutrophil-derived Activin-A moderates their pro-NETotic activity and attenuates collateral tissue damage caused by Influenza A virus infection
Source: Front Immunol. 2024 Feb 26;15:1302489. doi: 10.3389/fimmu.2024.1302489 (PMC10929267; doi:10.3389/fimmu.2024.1302489)
Supplement: Supplementary file 1 [file DataSheet_1.pdf]

frontiers

Supplementary Material

Neutrophil-derived Activin-A moderates their pro-NETotic activity and attenuates collateral tissue damage caused by Influenza A virus infection

Georgios Divolis*, Evgenia Synolaki, Athanasia Doulou, Ariana Gavriil, Christina C. Giannouli, Anastasia Apostolidou, Martyn L. Foster, Martin M. Matzuk, Panagiotis Skendros, Ioanna-Evdokia Galani and Paschalis Sideras

*** Correspondence:** Georgios Divolis: gdivolis@bioacademy.gr

1 Supplementary Figures and Tables

1.1 Supplementary Figures

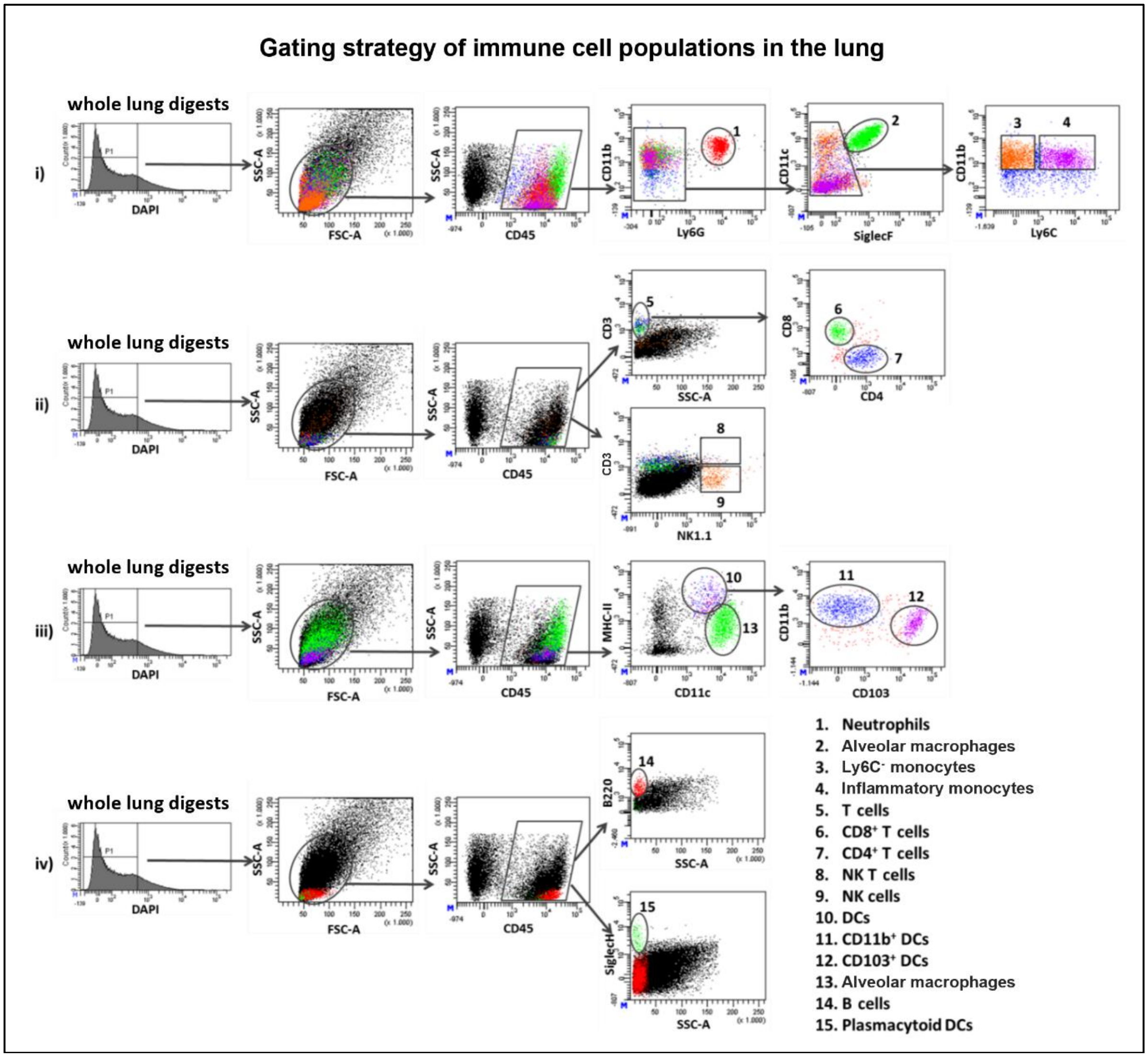

Supplementary Figure 1. Gating strategy followed for the characterization of immune cell populations in the lung. Flow cytometric profile for sorting **(i)** neutrophils, alveolar macrophages, Ly6C$^-$ and inflammatory monocytes, **(ii)** T cells (CD8$^+$, CD4$^+$, or NK), NK cells, **(iii)** conventional (CD11b$^+$ or CD103$^+$) DCs, **(iv)** B cells, and plasmacytoid DCs. DCs, dendritic cells; NK, natural killer cells.

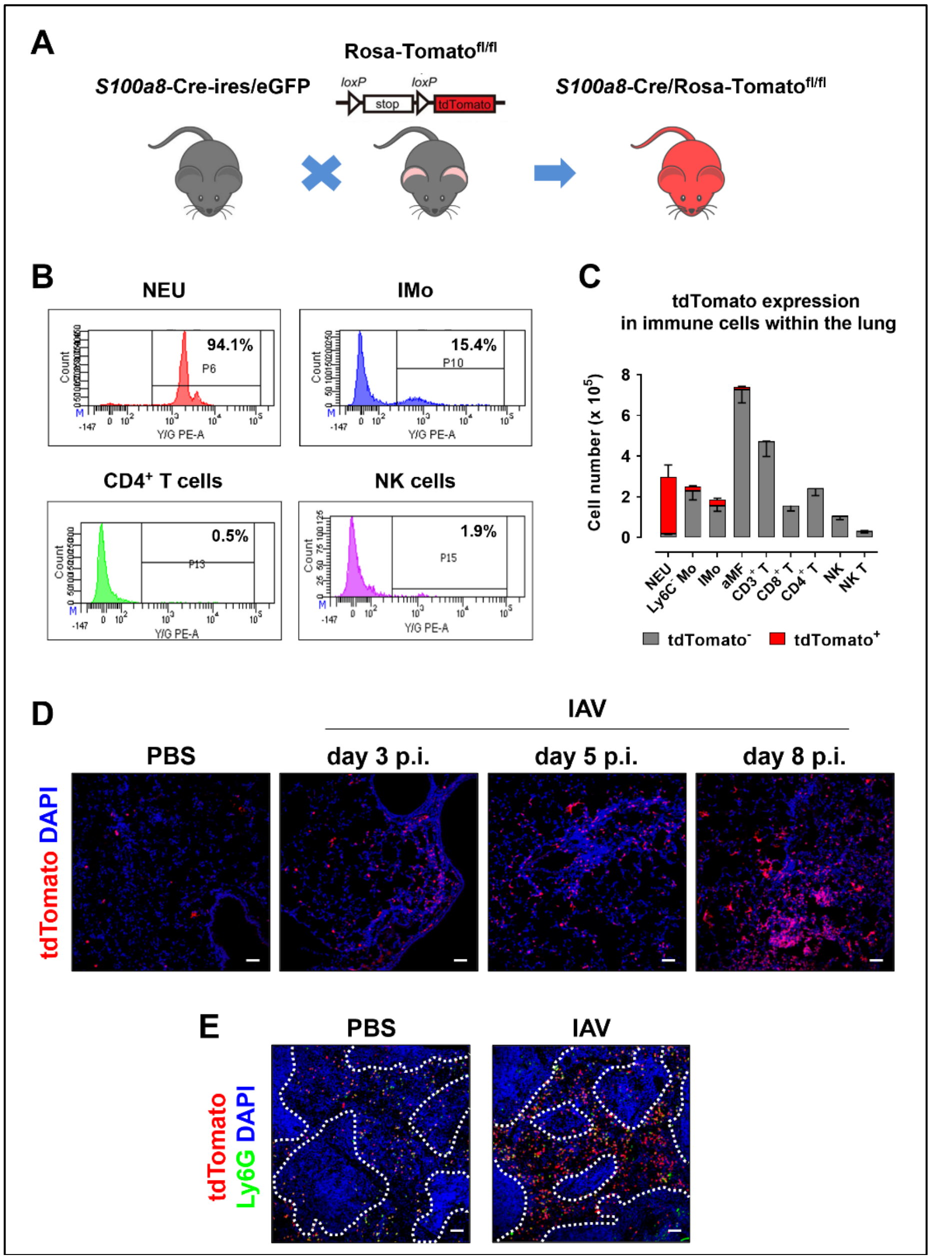

Supplementary Figure 2. Validation of specificity and efficiency of *S100a8*-Cre recombination, using *S100a8*-Cre/Rosa-Tomato$^{fl/fl}$ animals. (A) Schematic of experimental animals used in the study. *S100a8*-Cre-ires/eGFP were crossed with Rosa-Tomato$^{fl/fl}$ to generate *S100a8*-Cre/Rosa-Tomato$^{fl/fl}$ mice. **(B)** Representative histogram plots of tdTomato expression in neutrophils, inflammatory monocytes, CD4^{+} T cells, and NK cells in the lung of *S100a8*-Cre/Rosa-Tomato$^{fl/fl}$ animals. **(C)** Cell number of tdTomato^{+} and tdTomato^{-} immune cells within the lung of *S100a8*-Cre/Rosa-Tomato$^{fl/fl}$ animals, following flow cytometric analysis. Data are expressed as mean ± SEM of 3-6 animals/group from two independent experiments. **(D)** Representative confocal images for tdTomato (red) in lung sections of PBS- or IAV-treated *S100a8*-Cre/Rosa-Tomato$^{fl/fl}$ mice. Scale bars, 50 µm. (**E**) Representative confocal images for tdTomato (red) and Ly6G (green) in spleen cryosections of PBS- or IAV-treated *S100a8*-Cre/Rosa-Tomato$^{fl/fl}$ mice, at 8 days p.i. Scale bars, 50 µm. Dotted lines separate the white from the red pulp of the spleen. DAPI (blue) was used for nuclear staining. aMF, alveolar macrophages; IAV, Influenza A virus; IMo, inflammatory monocytes; Ly6C^{-} Mo, Ly6C^{-} monocytes; NEU, neutrophils; NK, natural killer cells; p.i., post-infection.

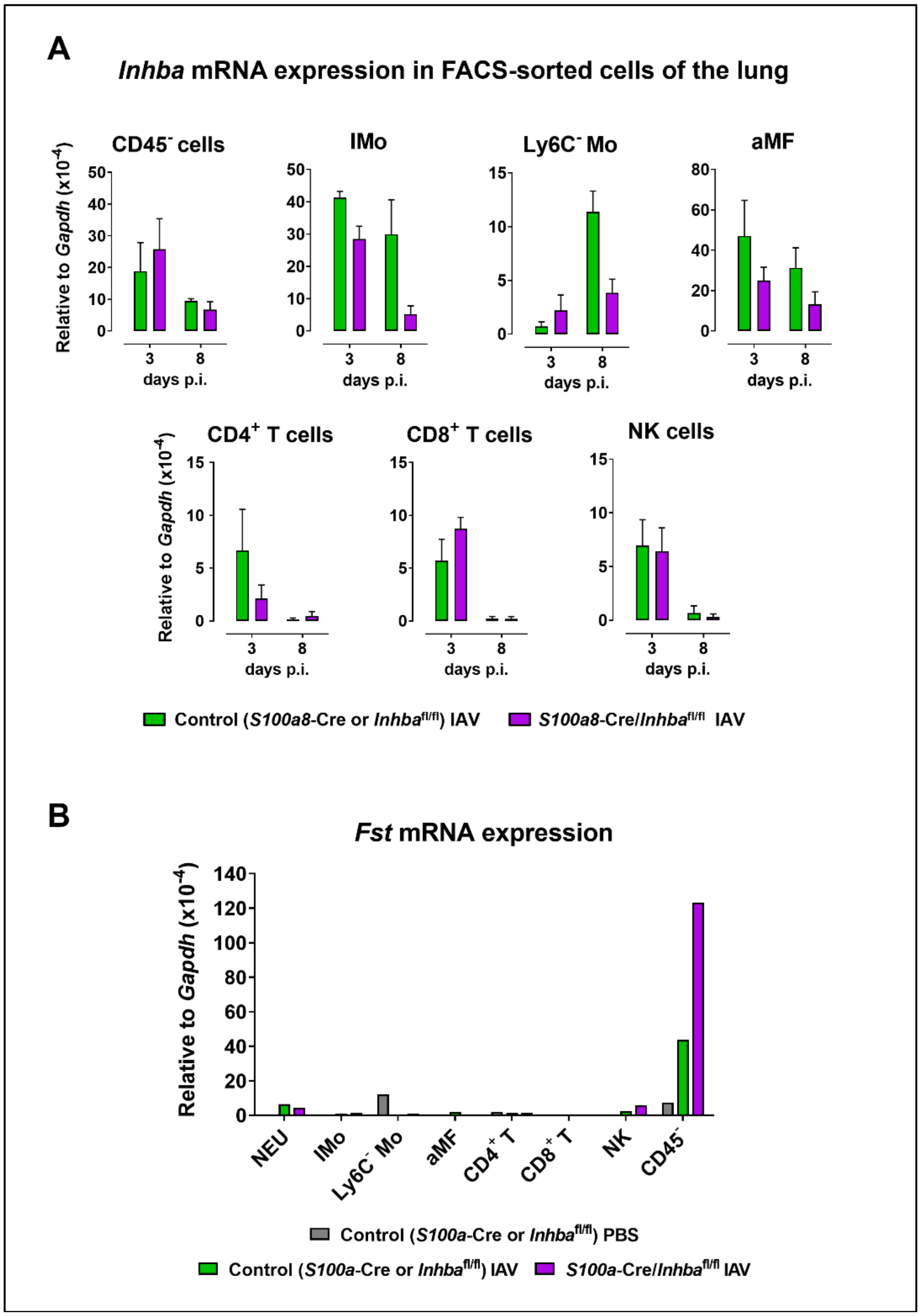

Supplementary Figure 3. *Inhba* and *Fst* mRNA expression in different FACS-sorted cell populations from the lungs of IAV-infected animals. (A) RT-qPCR analysis of mRNA levels for *Inhba* in FACS-sorted cells from the lungs of IAV-infected control (*S100a8*-Cre or *Inhba*$^{fl/fl}$) and *S100a8*-Cre/*Inhba*$^{fl/fl}$ mice, at 3 and 8 days p.i. Data are expressed as mean ± SEM of 3-4 animals/group. Nonparametric Mann-Whitney test was applied, and no statistical significance was found. **(B)** RT-qPCR analysis of mRNA levels for *Fst* in FACS-sorted cells from the lungs of PBS-treated and IAV-infected control and *S100a8*-Cre/*Inhba*$^{fl/fl}$ mice, at 8 days p.i. Pooled cDNA samples from three animals/group were used. aMF, alveolar macrophages; FACS, fluorescence-activated cell sorting; IAV, Influenza A virus; IMo, inflammatory monocytes; Ly6C^{-}Mo, Ly6C^{-} monocytes; NEU, neutrophils; NK, natural killer cells; p.i., post-infection.

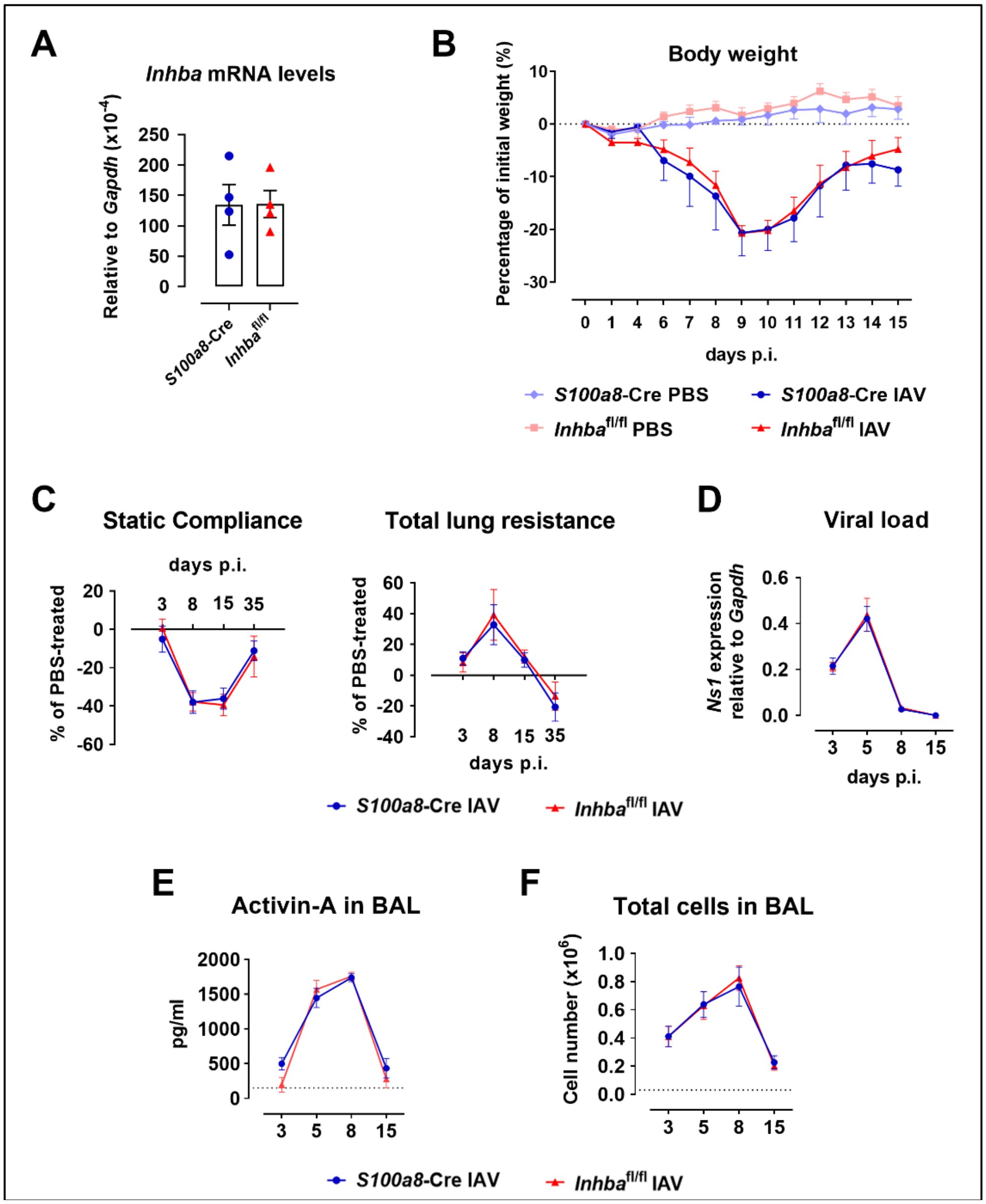

Supplementary Figure 4. The parental strains *S100a8*-Cre and *Inhba*$^{fl/fl}$ display similar phenotypes in response to IAV infection. **(A)** RT-qPCR analysis of mRNA levels for *Inhba* in FACS-sorted neutrophils infiltrating the lungs of IAV-infected *S100a8*-Cre and *Inhba*$^{fl/fl}$ mice, at 3 or 8 days p.i. Data are expressed as mean ± SEM of 4 animals/group. **(B)** Body weight expressed as percentage of the initial weight (day 0) of PBS-treated and IAV-infected (50 pfu) *S100a8*-Cre and *Inhba*$^{fl/fl}$ mice. Data are expressed as mean ± SEM of 4-8 animals/group. **(C)** Baseline-corrected static compliance and total lung resistance of IAV-infected *S100a8*-Cre and *Inhba*$^{fl/fl}$ mice. **(D)** Viral load in the lungs of IAV-infected *S100a8*-Cre and *Inhba*$^{fl/fl}$ mice, assessed by RT-qPCR analysis of *Ns1* viral gene expression. **(E)** Activin-A levels in the BAL of IAV-infected *S100a8*-Cre and *Inhba*$^{fl/fl}$ mice, measured by ELISA. **(F)** Number of total leukocytes in the BAL of IAV-infected *S100a8*-Cre and *Inhba*$^{fl/fl}$ mice. Dotted lines in **(E, F)** represent respective measurements in the BAL of PBS-treated animals. Data in **(C-F)** are expressed as mean ± SEM of 3-12 animals/group from two to three independent experiments. Nonparametric Mann-Whitney test was applied to all panels, and no statistical significance was found. BAL, bronchoalveolar lavage fluid; IAV, Influenza A virus; p.i., post-infection.

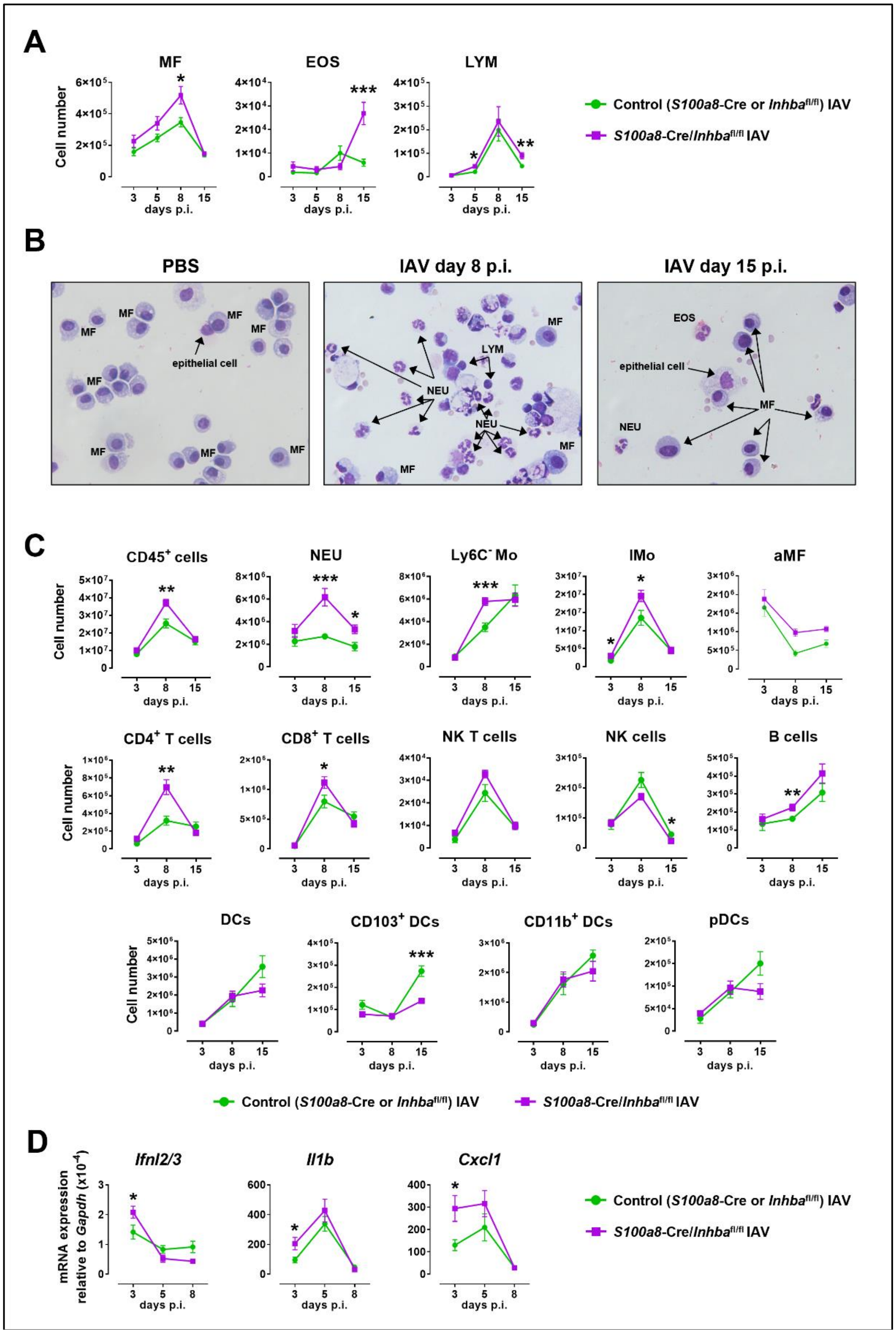

Supplementary Figure 5. Characterization of inflammatory cells in the BAL and lungs of control and *S100a8*-Cre/*Inhba*$^{fl/fl}$ animals, following IAV infection. (A) Numbers of macrophages, eosinophils, and lymphocytes in the BAL of IAV-infected control (*S100a8*-Cre or *Inhba*$^{fl/fl}$) and *S100a8*-Cre/*Inhba*$^{fl/fl}$ mice, as determined by May-Grünwald-Giemsa staining of cytospins. Data are

expressed as mean ± SEM of 7-19 animals/group. **(B)** Representative images of May-Grünwald-Giemsa-stained cells in the BAL of PBS-treated and IAV-infected *S100a8*-Cre/*Inhba*$^{fl/fl}$ mice at 8, and 15 days p.i. **(C)** Numbers of inflammatory cells infiltrating the lungs, as analyzed by flow cytometry in IAV-infected control and *S100a8*-Cre/*Inhba*$^{fl/fl}$ mice. Data are expressed as mean ± SEM of 6-11 animals/group. **(D)** RT-qPCR analysis of mRNA levels for *Ifnl2/3*, *Il1b*, and *Cxcl1* in the lungs of IAV-infected control and *S100a8*-Cre/*Inhba*$^{fl/fl}$ mice. Data are expressed as mean ± SEM of 3-9 animals/group. Nonparametric Mann-Whitney test was used in all panels, *p < 0.05, **p < 0.01, and ***p < 0.001. (a)MF, (alveolar) macrophages; BAL, bronchoalveolar lavage; EOS, eosinophils; IAV, Influenza A virus; IMo, inflammatory monocytes; Ly6C^{-} Mo, Ly6C^{-}monocytes; LYM, lymphocytes; NEU, neutrophils; NK, natural killer cells; (p)DCs, (plasmacytoid) dendritic cells; p.i., post-infection.

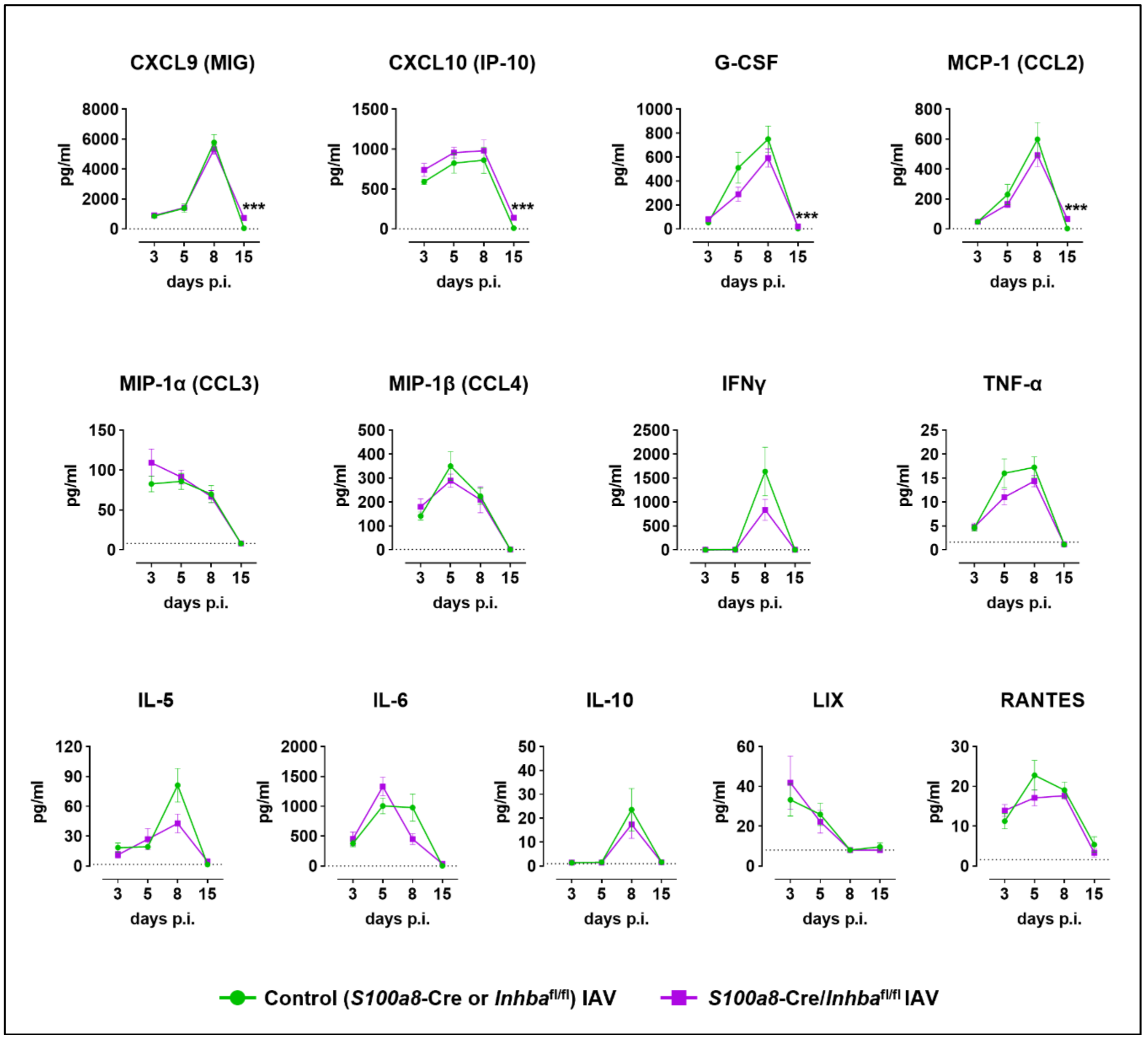

Supplementary Figure 6. Concentration of inflammatory cytokines and chemokines in the BAL of IAV-infected animals. Cytokines and chemokines in the bronchoalveolar lavage fluid (BAL) of IAV-infected control (*S100a8*-Cre or *Inhba*$^{fl/fl}$) and *S100a8*-Cre/*Inhba*$^{fl/fl}$ mice were analyzed using Milliplex technology. Dotted lines represent cytokine or chemokine levels in the BAL of PBS-treated animals. Data are expressed as mean ± SEM of 7-10 animals/group. Nonparametric Mann-Whitney test was applied, ***p < 0.001. IAV, Influenza A virus; p.i., post-infection.

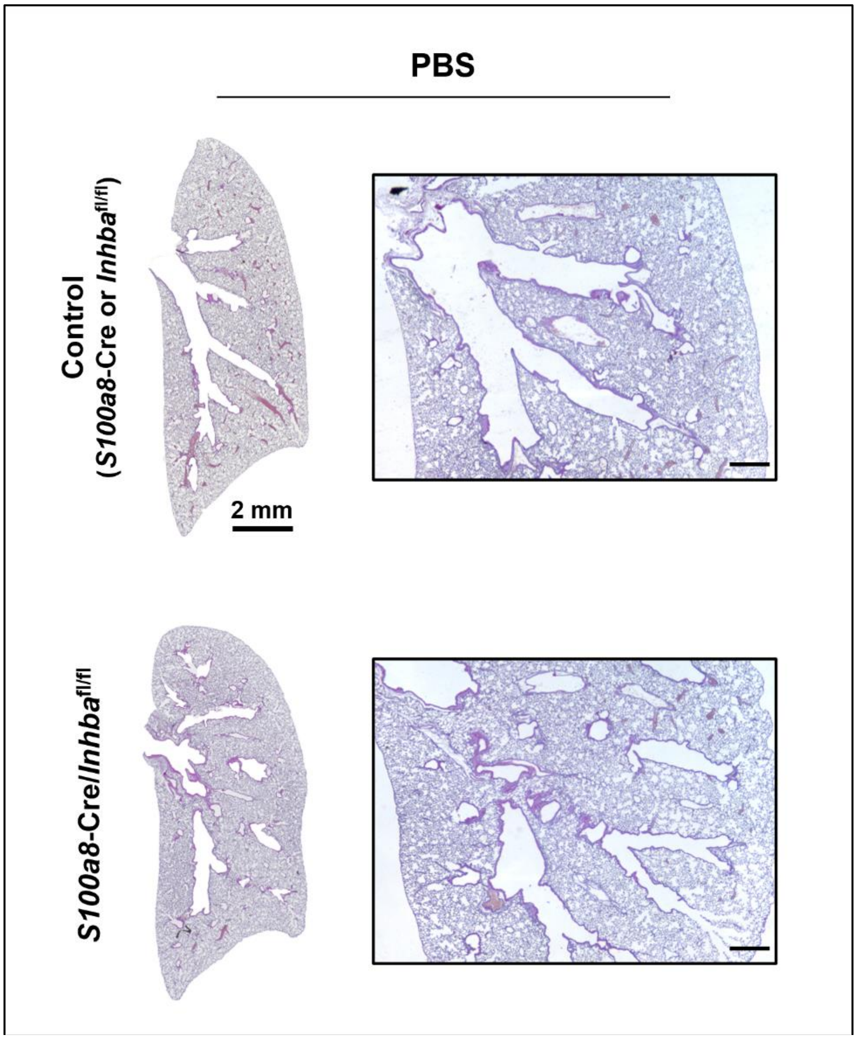

Supplementary Figure 7. Representative whole lung sections (Scale bar, 2 mm) and higher magnification images (Scale bars, 300 µm) of PBS-treated (uninfected) control (*S100a8*-Cre or *Inhba*$^{fl/fl}$) and *S100a8*-Cre/*Inhba*$^{fl/fl}$ mice, stained with hematoxylin and eosin (H&E).

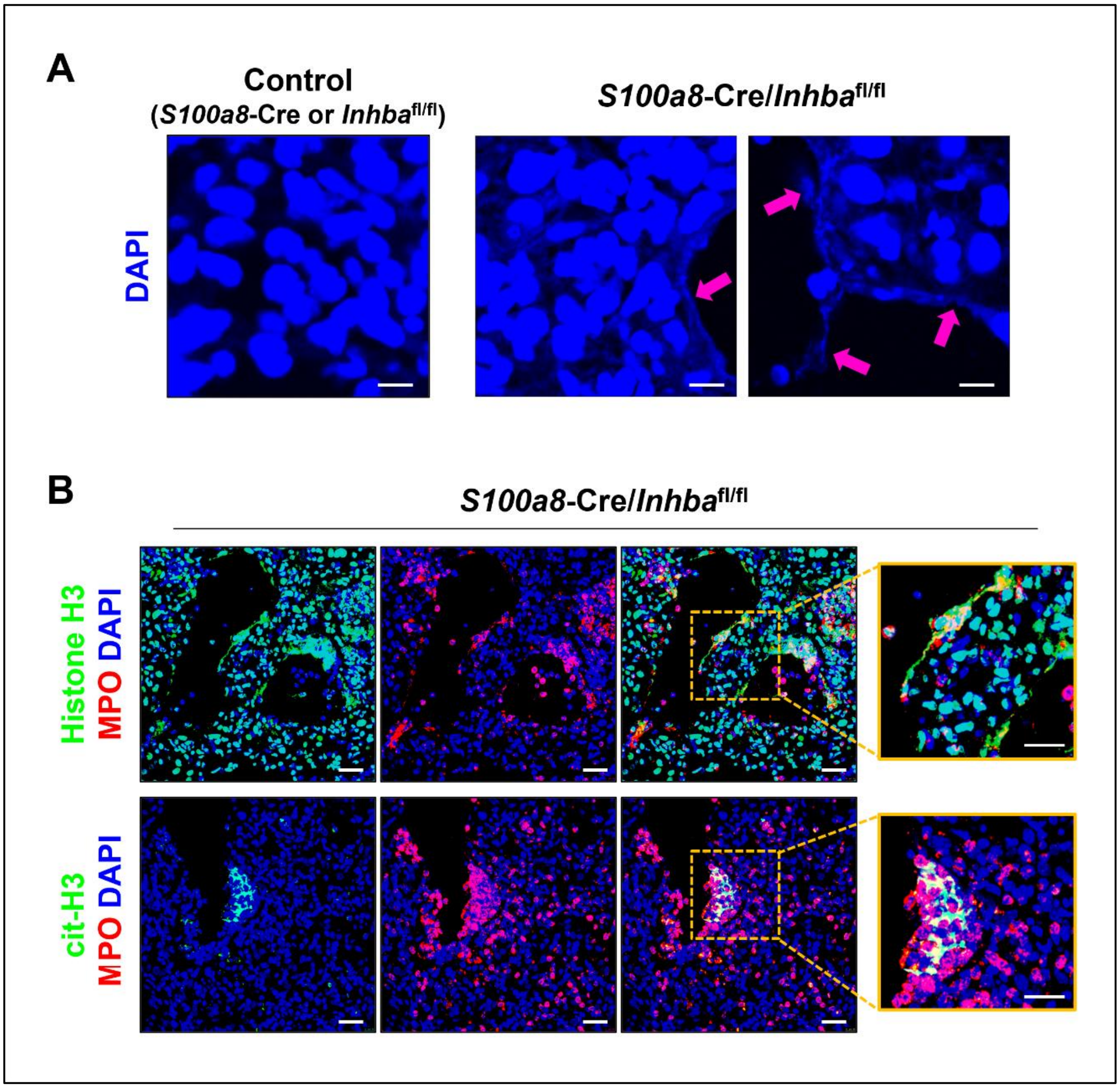

Supplementary Figure 8. Animals with Activin-A-deficient neutrophils are characterized by widespread NETosis in their lungs following IAV infection. (A) Representative immunofluorescence images of lung sections of IAV-infected control (*S100a8*-Cre or *Inhba*$^{fl/fl}$) and *S100a8*-Cre/*Inhba*$^{fl/fl}$ animals, at 15 days post-infection (p.i.), stained with DAPI (blue). Arrows show extracellular chromatin deposition within the injured tissues of IAV-infected *S100a8*-Cre/*Inhba*$^{fl/fl}$ animals. Scale bars, 7 μm. **(B)** Representative confocal immunofluorescence images for histone H3 (green, upper panels), cit-H3 (green, lower panels), MPO (red), and nuclei (DAPI, blue) in lung sections of IAV-infected *S100a8*-Cre/*Inhba*$^{fl/fl}$ mice, at 15 days p.i. Scale bars, 25 μm. Dashed squares indicate areas magnified on the right, showing colocalization of either histone H3 or cit-H3 with the neutrophil-specific marker MPO. cit-H3, citrullinated histone H3; IAV, Influenza A virus; MPO, myeloperoxidase.

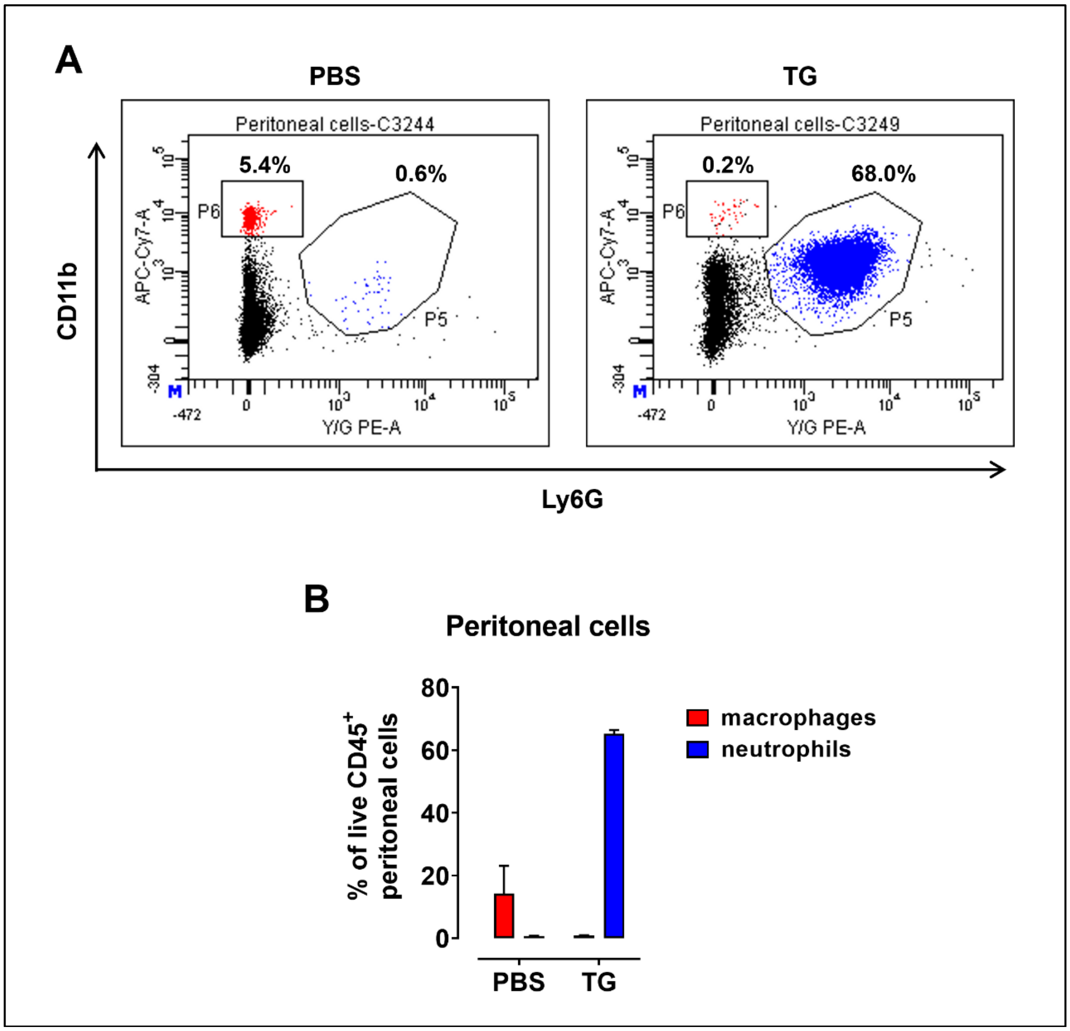

Supplementary Figure 9. Flow cytometric analysis of peritoneal cells isolated from PBS- and TG-injected animals. (A) Representative fluorescence-activated cell sorting (FACS) plots and **(B)** bar graph depicting the abundance of peritoneal neutrophils ($CD45^{+}CD11b^{+}Ly6G^{+}$) and macrophages ($CD45^{+}CD11b^{+}Ly6G^{-}$), expressed as % percentage of live $CD45^{+}$ peritoneal cells, in PBS-treated and TG-injected animals, six hours post-injection. Data in **(B)** are expressed as mean ± SEM of 3-18 animals/group. TG, thioglycollate.

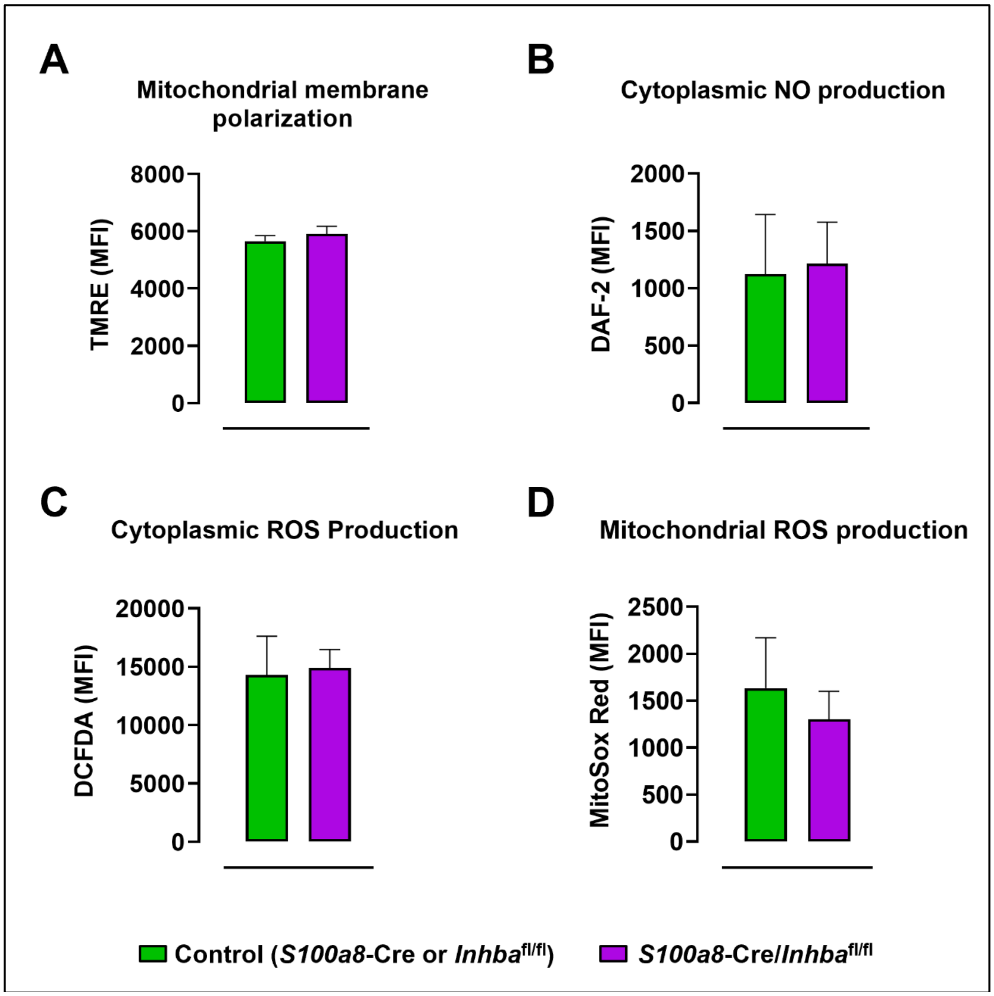

Supplementary Figure 10. Metabolic staining of peritoneal cells isolated from thioglycollate-injected animals. Control (*S100a8*-Cre or *Inhba*$^{fl/fl}$) and *S100a8*-Cre/*Inhba*$^{fl/fl}$ mice were intraperitoneally injected with thioglycollate, and six hours post-injection, peritoneal cells were isolated and stained with fluorescent metabolic probes. The MFI of fluorescent probes was evaluated by flow cytometric analysis within the neutrophil cell population ($CD45^+CD11b^+Ly6G^+$). **(A)** Alterations in mitochondrial membrane polarization were detected by TMRE perchlorate staining. **(B)** Cytoplasmic NO production was evaluated by DAF-2 staining. **(C, D)** Cytoplasmic and mitochondrial ROS production were detected using DCFDA **(C)** and MitoSox Red **(D)**, respectively. Data are expressed as mean ± SEM of 4 animals/group. Nonparametric Mann-Whitney test was used in all panels, and no statistical significance was found. DAF-2, 4,5-diaminofluorescein; DCFDA, 2′,7′-dichlorofluorescin diacetate; MFI, mean fluorescence intensity; NO, nitric oxide; ROS, reactive oxygen species; TMRE, tetramethyl rhodamine ethyl ester.

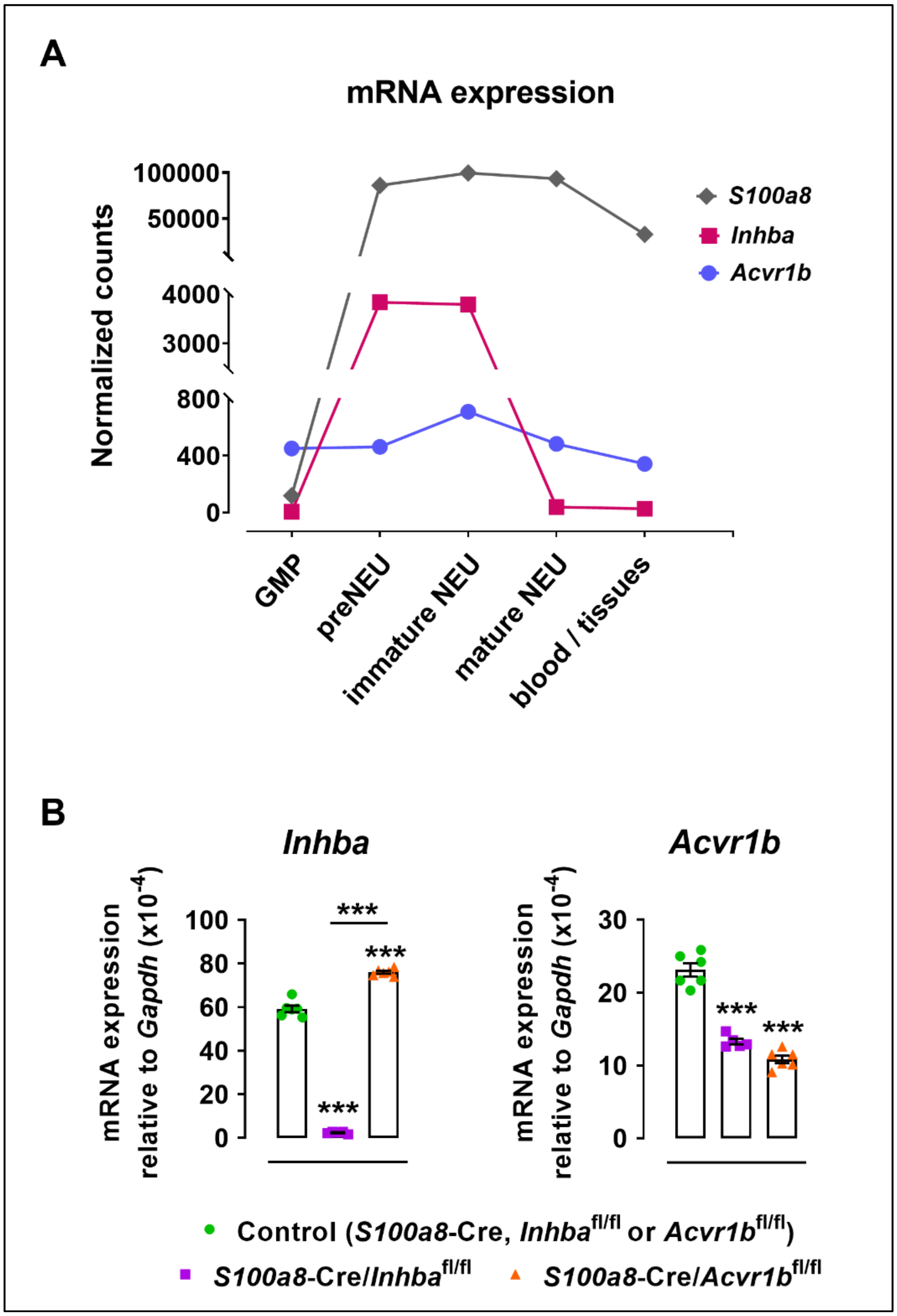

Supplementary Figure 11. *Inhba* and *Acvr1b* mRNA expression in bone marrow neutrophils. (A) Kinetics of mRNA expression of *S100a8, Inhba*, and *Acvr1b* during the maturation of neutrophils in the mouse bone marrow [adapted from (Evrard et al., 2018)]. **(B)** RT-qPCR analysis of mRNA levels for *Inhba* and *Acvr1b* in bone marrow cells isolated from control (*S100a8*-Cre, *Inhba*$^{fl/fl}$ or *Acvr1b*$^{fl/fl}$), *S100a8*-Cre/*Inhba*$^{fl/fl}$, and *S100a8*-Cre/*Acvr1b*$^{fl/fl}$ mice. Data are expressed as mean ± SEM of 5-6 animals/group. One-way ANOVA was applied, followed by Bonferroni's post hoc test, ***$p < 0.001$. GMP, granulocyte-monocyte progenitor; (pre) NEU, (pre-) neutrophils.

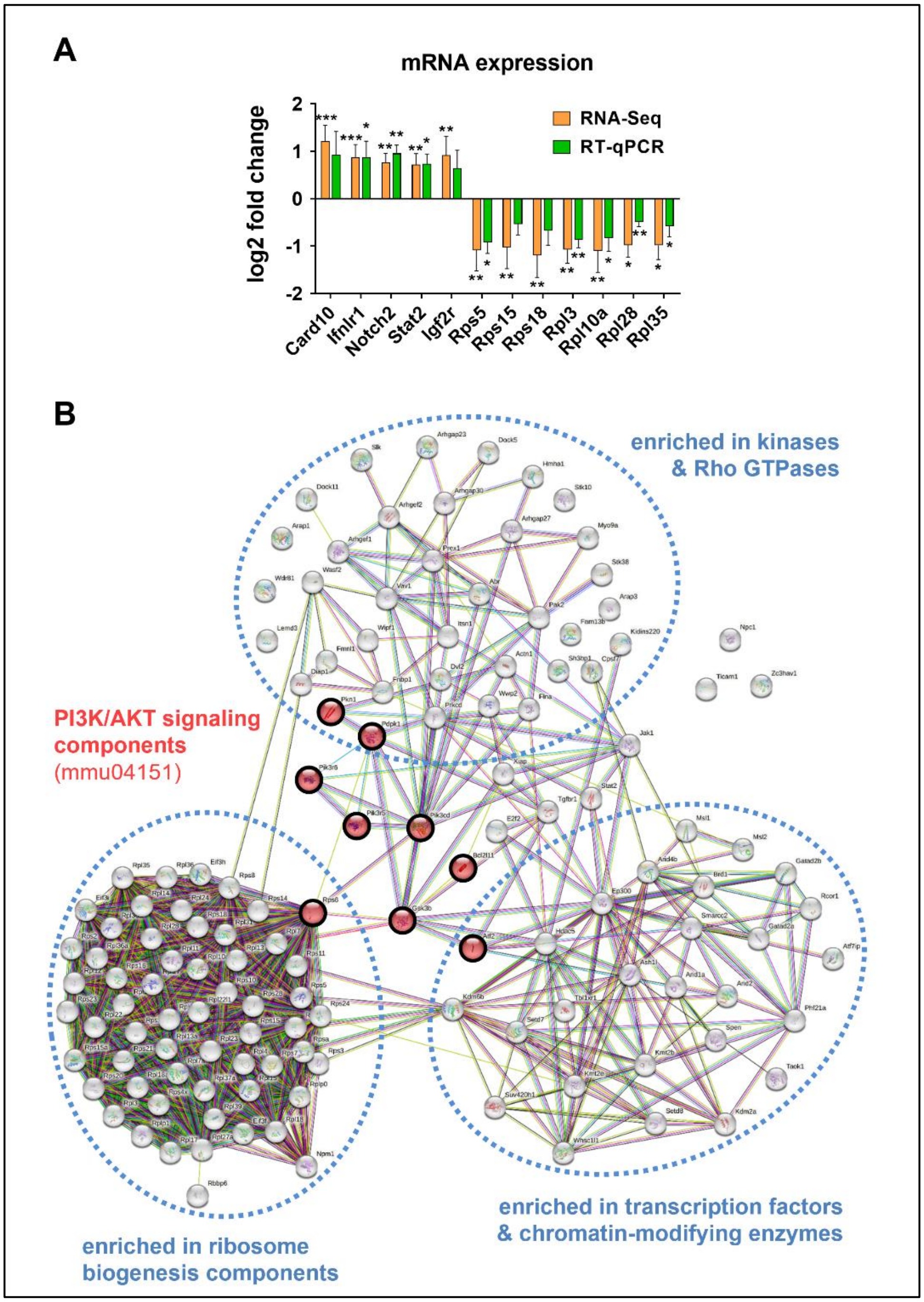

Supplementary Figure 12. Independent validation and network analysis of selected DEGs, derived from RNA-Seq analysis of Activin-A-deficient neutrophils. (A) Verification of selected up- and down-regulated DEGs, derived from RNA-Seq analysis of *S100a8*-Cre/*Inhba*$^{fl/fl}$ *versus* control (*S100a8*-Cre or *Inhba*$^{fl/fl}$) neutrophils, by RT-qPCR analysis of independently purified cells. Neutrophils were purified by cell sorting from the peritoneum of thioglycollate-injected animals, six hours post-injection. Data are expressed as mean ± SEM of 5-7 samples/group. Nonparametric Mann-Whitney test was applied to indicate significant differences compared to control neutrophils, *p < 0.05, **p < 0.01, and ***p < 0.001. **(B)** STRING network analysis of DEGs belonging to the top Ingenuity Pathway Analysis or Reactome pathways shown in **Figure 6D**, as determined by RNA-Seq analysis of control and *S100a8*-Cre/*Inhba*$^{fl/fl}$ neutrophils (6 samples/group). DEGs, differentially expressed genes; RNA-Seq, RNA-Sequencing; RT-qPCR, Real-Time quantitative PCR.

1.2 Supplementary Tables

Supplementary Table 1. Antibodies used for flow cytometry and cell sorting.

**Monoclonal anti-mouse antibody**	**Host species**	**Clone**	**Source**	**Cat. No.**
anti-**CD103**, PE-conjugated	Armenian hamster	2E7	BioLegend	121406
anti-**CD103**, PerCP/Cy5.5-conjugated	Armenian hamster	2E7	BioLegend	121415
anti-**CD11b**, APC/Cy7-conjugated	rat	M1/70	BioLegend	101226
anti-**CD11c**, PE/Cy7-conjugated	Armenian hamster	N418	BioLegend	117317
anti-**CD170/Siglec F**, BV421-conjugated	rat	S17007L	BioLegend	155509
anti-**CD170/Siglec F**, PerCP/Cy5.5-conjugated	rat	E50-2440	BD Pharmingen	565526
anti-**CD3e**, PE/Cy7-conjugated	Armenian hamster	145-2C11	BioLegend	100320
anti-**CD4**, FITC-conjugated	rat	GK1.5	BioLegend	100406
anti-**CD45**, APC-conjugated	rat	30-F11	BioLegend	103111
anti-**CD45R/B220**, BV510-conjugated	rat	RA3-6B2	BioLegend	103247
anti-**CD45R/B220**, FITC-conjugated	rat	RA3-6B2	BioLegend	103206
anti-**CD8a**, PerCP/Cy5.5-conjugated	rat	53-6.7	BioLegend	100734
anti-**Ly6C**, FITC-conjugated	rat	HK1.4	BioLegend	128006
anti-**Ly6G**, PE-conjugated	rat	1A8	BioLegend	127608
anti-**Ly6G**, PerCP/Cy5.5-conjugated	rat	1A8	BioLegend	127615
anti-**MHC-II** (I-A/I-E), FITC-conjugated	rat	M5/114.15.2	BioLegend	107605
anti-**NK1.1**, APC/Cy7-conjugated	mouse	PK136	BioLegend	108723
anti-**NK1.1**, BV510-conjugated	mouse	PK136	BioLegend	108737
anti-**NK1.1**, PE-conjugated	mouse	PK136	BioLegend	108708
anti-**Siglec H**, PE-conjugated	rat	551	BioLegend	129606

Supplementary Table 2. Primer pairs used for RT-qPCR analysis.

Target	Forward primer (5´→3´)	Reverse primer (5´→3´)	Product length (bp)	Tm (ºC)
Acvr1b	CACTGACACCATAGACATTGC	GATTGTCTCGTCAAGGACTTC	82	56
Card10	CCTGGACTTCCTCAATAGGTCT	ATCTGGACTCTTATCCTGGGG	88	59
Cxcl1	ATTGTCCAAAAGATGCTAAAAGG	TGTATAGTGTTGTCAGAAGCC	105	56
Fst	CGACAATACTCTCTTCAAGTGG	GGGTTTATTCTTCTTGTTCATTCG	130	59
Gapdh	CCAGTATGACTCCACTCACG	CTCCTGGAAGATGGTGATGG	97	56/59
Ifnl2/3	AGTGGAAGCAAAGGATTG	GAGATGAGGTGGGAACTG	138	56
Ifnlr1	GACGAGTACAGGCAGCTTCC	AGCATTGACCCTTAGGATCTTCTC	137	61
Igf2r	TGACAGAGGCACAGCATTCA	CCCTCGTGTGGAGTCAATGT	100	59
Il1b	CAACCAACAAGTGATATTCTCCATG	GATCCACACTCTCCAGCTGCA	152	59
Inhba	ACCTCGGAGATCATCACCTTTG	GCACGCTCCACTACTGACAG	101	59
Notch2	TCTCACCCCTGCTTTGTGTC	TCTGTCCACTGACACTGCTTC	122	59
**PR8 *Ns1***	TGTCAAGCTTTCAGGTAGATTG	CTCTTAGGGATTTCTGATCTC	117	59
Rpl10a	TTGGTCAAGAAGCTGGCTAAGA	CAGGATACGTGGGATCTGCTT	78	59
Rpl28	TCTGAAAGCCCGAAACTCCT	AGGTTTTCGCTGACCGGAT	127	59
Rpl3	TGGTCAAGGCTACCTCATCA	TGCACAAAGCCACCCAGT	105	59
Rpl35	GCCCGAGTCCTCACTGTTAT	GGCTTGTATTTCTTGCCCTTGT	77	59
Rps15	AGGTGGAGATCAAACCAGAGA	GGCTACTTGAGGGGGATGAA	130	59
Rps18	CACTTTTGGGGCCTTCGTG	AGGCCCAGAGACTCATTTCTT	99	59
Rps5	CGGAACATCAAGACCATCGC	TAGGAATTGGAGGAGCCCTTG	74	59
Stat2	TCAGACTTACCAGGCTTCCG	ACCAGAGTCAAGAAGCCGAA	116	59

2 References

Evrard, M., Kwok, I.W.H., Chong, S.Z., Teng, K.W.W., Becht, E., Chen, J., Sieow, J.L., Penny, H.L., Ching, G.C., Devi, S., Adrover, J.M., Li, J.L.Y., Liong, K.H., Tan, L., Poon, Z., Foo, S., Chua, J.W., Su, I.H., Balabanian, K., Bachelerie, F., Biswas, S.K., Larbi, A., Hwang, W.Y.K., Madan, V., Koeffler, H.P., Wong, S.C., Newell, E.W., Hidalgo, A., Ginhoux, F., and Ng, L.G. (2018). Developmental Analysis of Bone Marrow Neutrophils Reveals Populations Specialized in Expansion, Trafficking, and Effector Functions. *Immunity* 48**,** 364-379 e368.
